# ﻿A review of *Microdytes* J. Balfour-Browne, 1946 from Thailand, Laos, and Cambodia with descriptions of five new species and new records (Coleoptera, Dytiscidae)

**DOI:** 10.3897/zookeys.1159.99218

**Published:** 2023-04-25

**Authors:** Ryohei Okada, Weeyawat Jaitrong, Günther Wewalka

**Affiliations:** 1 Thailand Natural History Museum, National Science Museum, Technopolis, Pathum Thani, Thailand Thailand Natural History Museum, National Science Museum Pathum Thani Thailand; 2 Coleopterological Society of Japan, National Museum of Nature and Science, Tsukuba, Japan Coleopterological Society of Japan, National Museum of Nature and Science Tsukuba Japan; 3 Starkfriedgasse 16/1/3, A – 1190 Vienna, Austria Unaffiliated Vienna Austria

**Keywords:** Diving beetles, faunistics, key to species, Southeast Asia, taxonomy, zoogeography

## Abstract

The diving beetle genus *Microdytes* J. Balfour-Browne, 1946 in Thailand, Laos, and Cambodia is reviewed, and five new species are described: *Microdyteseliasi* Wewalka & Okada, **sp. nov.** (Thailand, Cambodia), *M.jeenthongi* Okada & Wewalka, **sp. nov.** (Thailand), *M.maximiliani* Wewalka & Okada, **sp. nov.** (Laos, China), *M.sekaensis* Okada & Wewalka, **sp. nov.** (Thailand, Laos), *M.ubonensis* Okada & Wewalka, **sp. nov.** (Thailand, Laos). Two species are the first country records: *M.balkei* Wewalka, 1997 (Laos, Cambodia) and *M.wewalkai* Bian & Ji, 2009 (Laos). For 12 and 8 species, the first provincial records from Thailand and Laos, respectively, are given. A checklist, a key to the 25 known *Microdytes* species from these countries, and habitus images and illustrations of diagnostic characters are provided. Distribution maps of the recorded species are presented, and species distribution patterns are also briefly discussed.

## ﻿Introduction

The dytiscid genus *Microdytes* J. Balfour-Browne, 1946 contains 47 described species classified in the tribe Hyphydrini of the subfamily Hydroporinae (Nilsson and Hájek 2023). These minute diving beetles (adult length up to 2.3 mm) generally occur in small seeps, springs, and streams, and a few are also known to inhabit subterranean and hygropetric environments (Wewalka 1997; Wewalka et al. 2007; Sheth et al. 2020). *Microdytes* species occur throughout southern and southeastern Asia from Nepal to southern India to southern China, southern Japan, Philippines, and Indonesia (Miller and Wewalka 2010). So far, 16 species were recorded from Thailand, and this country was regarded as the center of distribution of the genus by Wewalka (1997, 2011).

During 2018–2022, as part of the field survey by the senior author to reveal diversity of Thailand diving beetles, a total of 228 *Microdytes* specimens were collected from 30 localities. For comparative purposes, we also studied specimens from Thailand, Laos, and Cambodia, mainly collected as part of the “NHMB Basel, Laos Expeditions” in 2011 and 2012 (Geiser and Nagel 2013).

In this paper, we describe five new species from Thailand, Laos, and Cambodia, increasing the number of known *Microdytes* species to 52. New and first regional records from those countries are also given. A checklist and a key to all *Microdytes* species known from these countries are provided. To facilitate the identification of the species, photographs of habitus and discriminant characters are provided for the first time. Distribution maps of the recorded *Microdytes* species are also presented, and their distribution patterns and microhabitat preferences are briefly discussed.

## ﻿Materials and methods

The study was based on the examination of 695 specimens: 241 from Thailand, 420 from Laos, and 34 from Cambodia. These specimens are deposited in the following institutions and private collections:

**CGW** Collection Günther Wewalka, Vienna, Austria;

**CRO** Collection Ryohei Okada, Tokyo, Japan;

**MNHN**Muséum National d’Histoire Naturelle, Paris, France;

**NMB**Naturhistorisches Museum, Basel, Switzerland;

**NMP**National Museum (Natural History), Prague, Czech Republic;

**NMW**Naturhistorisches Museum Wien, Vienna, Austria;

**THNHM** Thailand Natural History Museum, Pathum Thani, Thailand.

Beetles were pin mounted on square or triangular card points. Male genitalia were dissected, then put on cards for detailed observation. The holotypes, paratypes, and lectotype of the closely related species were examined. Specimens from Myanmar were also used for comparisons.

The beetles were studied with an Olympus SZX10 compound microscope equipped with Nomarsky optics up to 1000×. Habitus photographs were taken using a Canon EOS 7D Mark II digital camera with attached Canon MP-E65 mm f/2.8 macro lens with 5:1 optics. Male genitalia were illustrated wet, using an Olympus BHT transmitted light microscope with a RICOH GX6 attachment. Composite images were created using the image stacking software Helicon Focus (Helicon Soft Ltd., Kharkov), and subsequently edited with Adobe Photoshop elements (2008) (Adobe Systems Inc., USA) where necessary.

The terminology to denote the orientation of the genitalia follows Miller and Nilsson (2003); the style of the descriptive notes follows Wewalka (1997, 2011) and Miller and Wewalka (2010).

Body measurements were made with a compound microscope equipped with a micrometer eyepiece. The abbreviations of measurements used in this study are as follows: **TL** (total body length), TL-H (body length without head), MW (maximum width of body). The ratio **TL/MW** was also calculated. Measurements of holotypes are added between round brackets. Label data of holotype specimens are cited between quotation marks. A backslash (\) indicates separate labels. Our comments are given between square brackets.

**Figure 1.**
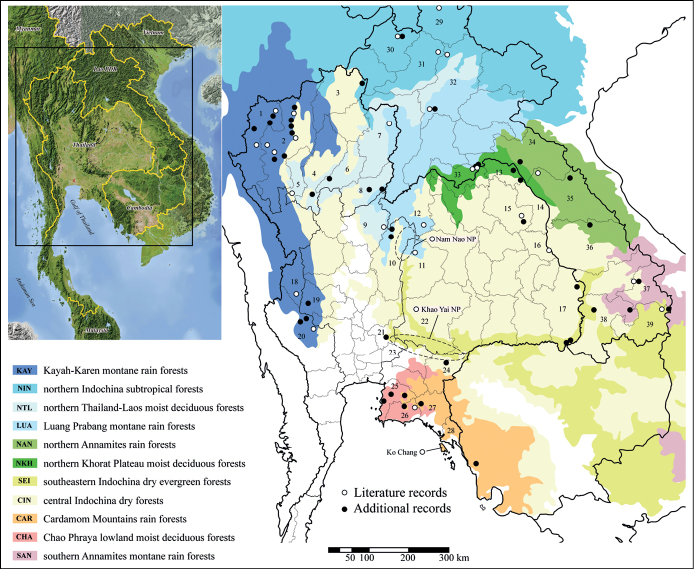
Known localities of *Microdytes* species in Thailand, Laos, and Cambodia, including literature records, sampling sites and additional records in this study. For those records where specific information of province was not reported, the location was mapped by dotted lines. Ecoregion classification is given following Olson et al. (2001). Thailand: **1** Mae Hong Son **2** Chiang Mai **3** Chiang Rai **4** Lampang **5** Lamphun **6** Phrae **7** Nan **8** Uttaradit **9** Phitsanulok **10** Phetchabun **11** Chaiyaphum **12** Loei **13** Bueng Kan **14** Nakhon Phanom **15** Sakon Nakhon **16** Mukdahan **17** Ubon Ratchathani **18** Tak **19** Uthai Thani **20** Kanchanaburi **21** Saraburi **22** Nakhon Ratchasima **23** Nakhon Nayok **24** Sa Kaeo **25** Chonburi **26** Rayong **27** Chanthaburi **28** Trat. Laos: **29** Phongsaly **30** Luang Namtha **31** Oudomxay **32** Luang Prabang **33** Vientiane **34** Bolikhamxai **35** Khammouane **36** Savannakhet **37** Sekong **38** Champasak **39** Attapeu.

All localities where *Microdytes* species were recorded in Thailand, Laos, and Cambodia are shown in Fig. 1. Literature records (Wewalka 1997, 2011) and additional records in this study are symbolized separately. For interpretation of distribution patterns of *Microdytes*, we mapped the recorded localities onto terrestrial ecoregions as defined by Olson et al. (2001). The main ecoregions for the distributions of species in Thailand, Laos, and Cambodia are abbreviated as follows:

**T** Thailand;

**L** Laos;

**C** Cambodia;

**KAY** Kayah-Karen montane rain forest;

**NIN** northern Indochina subtropical forests;

**LUA** Luang Prabang montane rain forests;

**NAN** northern Annamites rain forests;

**NKH** northern Khorat Plateau moist deciduous forests;

**SEI** southeastern Indochina dry evergreen forests;

**CIN** central Indochina dry forests;

**CAR** Cardamom Mountains rain forests;

**CHA** Chao Phraya lowland moist deciduous forests.

## ﻿Results

### ﻿Descriptions

#### ﻿ Microdytes eliasi

Taxon classificationAnimaliaColeopteraDytiscidae

Wewalka & Okada sp. nov.

E3744614-B5C2-5BF8-9856-06CE116951EF

https://zoobank.org/8C12C23B-3B90-4F46-9D48-F5ABD6625734

**Figures 2–6.**
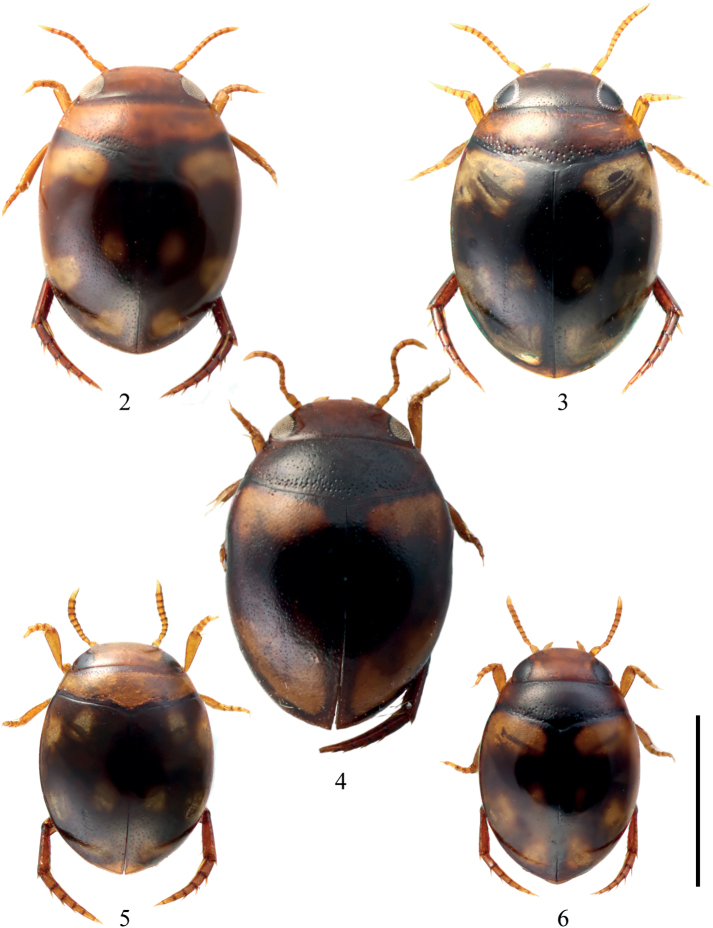
Habitus of **2***Microdyteseliasi* sp. nov., female, paratype **3***M.jeenthongi* sp. nov., male, holotype **4***M.maximiliani* sp. nov., male, holotype **5***M.sekaensis* sp. nov., male, holotype **6***M.ubonensis* sp. nov., male, holotype. Scale bar: 1.0 mm.

**Figures 7–11.**
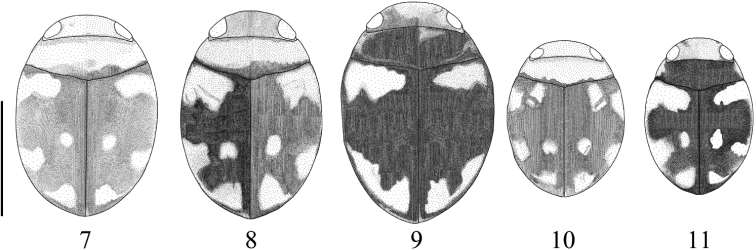
Variation of markings of **7***Microdyteseliasi* sp. nov. **8***M.jeenthongi* sp. nov **9***M.maximiliani* sp. nov. **10***M.sekaensis* sp. nov. **11***M.ubonensis* sp. nov. Scale bar: 1.0 mm.

**Figures 12–14.**
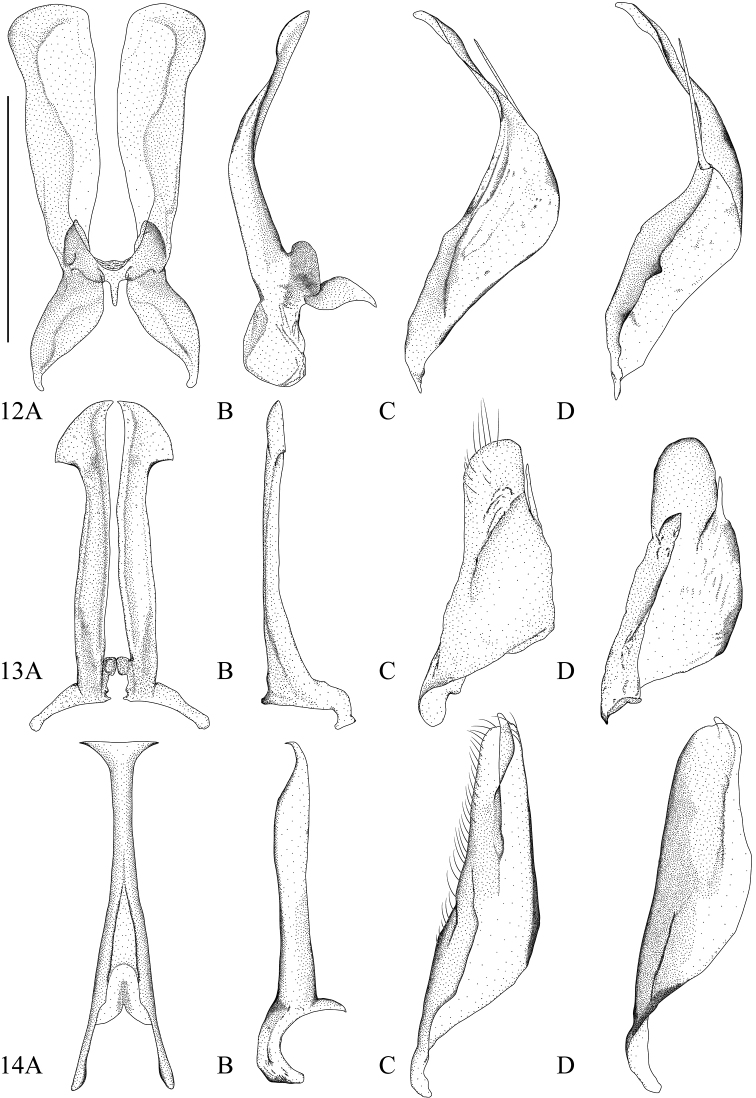
**12***Microdyteseliasi* sp. nov. **13***M.jeenthongi* sp. nov. **14***M.maximiliani* sp. nov. **A** median lobe in ventral aspect **B** median lobe in lateral aspect **C** left paramere in lateral aspect **D** right paramere in medial aspect. Scale bar: 0.25 mm.

Figs 2 , 7 , 12 , 42C , 43C

##### Type locality.

Cambodia, Koh Hong Province, 20 km SW Koh Hong, Tatai River, 11°34'N, 103°07'E, alt. 50–300 m.

##### Type material.

***Holotype*** (NMB): ♂ “Cambodia SW, Tatai riv. 20 km SE Koh Hong 11°34'N, 103°07'E, 3.–19.v.2005, 50–300 m, E. Jendek, O. Šauša leg.” [printed white label] \ “HOLOTYPUS Microdyteseliasi sp. nov. Wewalka & Okada 2022” [printed red label]. ***Paratypes***: (47 exs.): 7♂♂, 14♀♀, with same data as the holotype (CGW, CRO, NMB, NMP); 2♂♂, Thailand, Saraburi Province, Kaeng Khoi District, Ched Khot St. 122 (alt. 140 m), 14°28'26"N, 101°09'59"E, 30.V.2020, R. Okada leg. (CRO, THNHM); 4♂♂, 4♀♀, Thailand, Sa Kaeo Province, Mueang Sa Kaeo District, Ban Kaeng St. 277 (alt. 80 m), 13°58'47"N, 102°11'29"E, 30.I.2022, R. Okada leg. (CGW, CRO, THNHM); 2♂♂, 3♀♀, Thailand, Chonburi Province, Bo Thong District, That Thong St. 107 (alt. 100 m), 13°15'01"N, 101°22'34"E, 14.III.2020, R. Okada leg. (CGW, CRO, THNHM); 2♂♂, 2♀♀, same locality St. 298 (alt. 100 m), 13.VII.2022, R. Okada leg. (CGW, CRO); 1♀, same province, Si Racha District, Bang Phra St. 269 (alt. 90 m), 13°14'39"N, 101°02'20"E, 26.XII.2021, R. Okada leg. (CRO); 3♂♂, 2♀♀, Thailand, Chanthaburi Province, Khaeng Hong Maeo District, Kaeng Hong waterfall St. 257 (alt. 160 m), 13°02'57"N, 101°45'35"E, 26.VI.2021, R. Okada leg. (CGW, CRO, THNHM); 1♀, same district, Kha riv. St. 258 (alt. 90 m), 12°57'38"N, 101°46'30"E, 26.VI.2021, R. Okada leg. (CRO). All paratypes are provided with printed red paratype labels.

##### Diagnosis.

*Microdyteseliasi* sp. nov. very closely resembles *M.maculatus* (Motschulsky, 1860) (Figs 25, 41) in size and coloration but differs from this species by the more regularly oval habitus, the laterally expanded median lobe at apex and constricted tips of paramere (Figs 12, 42C, 43C) (see also comments in *M.maculatus*). It is also similar to *M.feryi* Wewalka, 2011 (Fig. 21) in habitus and coloration, but it is smaller and can be distinguished by male genitalia [compared with a male paratype from Myanmar, “Tenasserim, Birma Coll. V. Helfer National Museum Prague” (CGW)].

##### Description.

***Measurements*.**TL = 1.64–1.85 mm (1.76 mm), TL-H = 1.49–1.65 mm (1.55 mm), MW = 1.12–1.25 mm (1.24 mm), TL/MW = 1.33–1.44 (1.42). Body regularly oval, moderately convex (Fig. 2).

***Coloration*.** Head reddish brown. Pronotum reddish brown, narrowly dark brown along anterior and posterior margins. Elytron dark brown with yellowish brown markings forming a distinct transverse band near base not reaching suture connected along lateral margin with a post-median transverse lateral band, with a small post-median spot near suture, and a triangular spot near apex (Figs 2, 7). Ventral surface of head, prothorax and elytral epipleuron yellowish brown; thorax and abdominal ventrites reddish brown to dark brown. Legs, antennae and palpi yellowish brown.

***Sculpture and structure*.** Head finely, sparsely, and relatively regularly punctured; anterior half to two-thirds finely microreticulate; clypeus not bordered. Pronotum quite regularly, sparsely, and fairly strongly punctured, with coarser punctures along posterior margin; without microreticulation; lateral margins finely bordered, regularly rounded. Elytron quite regularly, moderately densely and fairly strongly punctured, progressively finer and sparser towards lateral margin; without longitudinal rows of stronger punctures; highly polished and shining; without microreticulation. Ventral surface: metacoxae and metasternum strongly but sparsely punctured, abdomen finely and sparsely punctured; without microreticulation.

**Male.** The two parts of the median lobe expanded laterally at apex in ventral aspect (Figs 12A, 42A); slightly curved in lateral aspect (Fig. 12B). The tips of parameres twisted in lateral aspect (Fig. 12C, D); constricted in ventral aspect (Fig. 43C).

**Female.** Without secondary sexual characters. Sclerotized spermatheca not found.

##### Variation.

Variation of markings is shown in Fig. 7.

##### Etymology.

This species is dedicated to Elias Bramböck, Vienna, Austria. The species epithet is a name in the genitive singular.

##### Habitat.

In Thailand, this species was collected in small streams at low altitude lower than 200 m (Fig. 45).

##### Distribution.

Thailand: Saraburi, Sa Kaeo, Chonburi, and Chanthaburi provinces; Cambodia: Koh Hong Province.

#### ﻿ Microdytes jeenthongi

Taxon classificationAnimaliaColeopteraDytiscidae

Okada & Wewalka sp. nov.

0A721176-1CF1-5222-95AA-7A35CFED94ED

https://zoobank.org/D3B3AE52-D18B-49E8-AAB7-E0DC2F5E6CD1

Figs 3 , 8 , 13 , 39E

##### Type locality.

Thailand, Chiang Mai Province, Mae Chaem District, Tha Pha, 18°30'46"N, 98°25'25"E, alt. 720 m.

##### Type material.

***Holotype*** (THNHM): ♂, “THAI: Chiang Mai Mae Chaem Dist., Tha Pha St. 166 (alt. 720 m) 15.VIII.2020” [printed white label] \ “HOLOTYPE Microdytesjeenthongi sp. nov. Okada & Wewalka 2022 [printed red label]”. ***Paratypes*** (3 exs.): 2♂♂, same data as the holotype (CGW, NMW); 1♂, same locality, 4.VII.2020, R Okada leg (CRO). All paratypes are provided with printed red paratype labels.

##### Diagnosis.

*Microdytesjeenthongi* sp. nov. resembles *M.shunichii* Satô, 1995 (Fig. 36) and *M.zetteli* Wewalka, 1997 (Fig. 37) in habitus, coloration and very finely punctured metasternum and metacoxae. From *M.shunichii* it differs by the reddish brown pronotum and very finely and sparsely punctured elytra. From *M.zetteli* it can be distinguished by larger size, less finely and sparsely punctured head.

##### Description.

***Measurements*.**TL = 1.77–1.82 mm (1.79 mm), TL-H = 1.23–1.26 mm (1.26 mm), MW = 1.18–1.23 mm (1.23 mm), TL/MW = 1.46–1.50 (1.49). Body regularly oval, moderately convex (Fig. 3).

***Coloration*.** Head reddish brown. Pronotum reddish brown, along the anterior margin narrowly and along the posterior margin more widely dark brown, especially medially. Elytron dark brown with a distinct transverse band at the base not reaching suture, continuing along the margin to a post-median transverse band and a triangular spot near the apex, and a small round post-median spot near suture (Figs 3, 8). Ventral surface of head and prothorax yellowish brown; thorax, abdominal ventrites and elytral epipleuron reddish brown to dark brown. Legs, antennae and palpi yellowish brown.

***Sculpture and structure*.** Head finely and sparsely, slightly irregularly punctured; anterior forth finely microreticulate; clypeus not bordered. Pronotum punctured moderately irregularly, finely, and sparsely, very coarsely along posterior margin; without microreticulation; lateral margins finely bordered, regularly rounded. Elytron very sparsely and finely punctured, progressively finer and sparser towards lateral margin; one longitudinal row of punctures distinct; highly polished and shining; without microreticulation. Ventral surface: metasternum and metacoxae very finely, sparsely, and irregularly punctured; abdomen almost without punctures; without microreticulation.

**Male.** The two parts of the median lobe expanded and pointed at apex forming the unique arrowhead shape in ventral aspect (Fig. 13A); almost straight and slightly expanded downwards at apex in lateral aspect (Fig. 13B). Parameres moderate triangular in lateral aspect (Figs 13C, D).

**Female.** Unknown.

##### Variation.

Variation of markings is shown in Fig. 8.

##### Etymology.

This species is dedicated to Tadsanai Jeenthong, collection manager of National Science Museum, Thailand. The species epithet is a name in the genitive singular.

##### Habitat.

This species was collected in a small, shallow stream with gravel bottom flowing through a small valley. In this stream, specimens were collected from a restricted point where tree roots were exposed along the river bottom (Fig. 46).

##### Distribution.

Thailand: Chiang Mai Province.

#### ﻿ Microdytes maximiliani

Taxon classificationAnimaliaColeopteraDytiscidae

Wewalka & Okada sp. nov.

B2456B3F-39C7-5B39-82D2-EEF6964E097D

https://zoobank.org/E1377AC3-9A63-4D0A-BEEF-FEEFA69E26F3

Figs 4 , 9 , 14 , 38

##### Type locality.

Laos, Louang Namtha Province, 10 km E Muang Sing, 21°09–10'N, 101°13–15'E, alt. 750–1400 m.

##### Type material.

***Holotype*** (NMB): ♂, “Laos, Louang Namtha prov., 10 km E Muang Sing, 750–1400 m, Ban Oudonsinh B. Nam Det \ B. Nam Mai, 21°09–10'N \ 101°13–15'E, 14.–20.V.2011” \ “NHMB Basel Laos 2011 Expedition D. Hauk & M. Geiser” [printed white labels] \ “HOLOTYPUS Microdytesmaximiliani sp. nov. Wewalka & Okada 2022” [printed red label]. ***Paratypes*** (4 exs.): 2♂♂, 1♀, with same data as the holotype (CGW, NMB, NMP); 1♀, China: Yunnan, Xishuangbanna, ca. 10 km NW Menglun, ca. 700–800 m, 7.XI.1999, M. Jäch et al. (CWBS 360) (NMW). All paratypes are provided with printed red paratype labels.

##### Diagnosis.

*Microdytesmaximiliani* sp. nov. resembles *M.satoi* Wewalka, 1997 in coloration of pronotum and elytra but differs from this species by darker head, larger size, a distinct impression on lateral side in anterior third of elytron (Fig. 38) and male genitalia. It is similar to *M.paoloi* Wewalka, 2011 in size and coloration of pronotum but can be distinguished by elytral markings, the impression on lateral side of elytron and male genitalia. The shape of median lobe of aedeagus of *M.maximiliani* is very similar to that of *M.schoenmanni* Wewalka, 1997, but this species is much smaller and differs in coloration, punctation and a missing impression on lateral side of elytron.

##### Description.

***Measurements*.**TL = 1.60–2.00 mm (1.86 mm), TL-h = 1.40–1.80 mm (1.61 mm), MW = 1.30–1.35 mm (1.30 mm), TL/MW = 1.23–1.43 (1.43). Body broad and regularly oval, quite convex (Figs 4, 38).

***Coloration*.** Head dark reddish brown, darker on vertex and along eyes. Pronotum dark brown. Elytron dark brown with yellowish brown markings forming a distinct transverse band near base not reaching suture and lateral margin, a post-median lateral spot often connected with a triangular spot near apex (Figs 4, 9). Elytral epipleuron and ventral surface dark brown to black. Legs, antennae and palpi reddish brown.

***Sculpture and structure*.** Head finely and sparsely punctured, stronger punctures along eyes and on vertex; with fine microreticulation missing on vertex; clypeus not bordered. Pronotum irregularly punctured with sparse fine punctures and strong punctures concentrated along posterior margin and sparsely on disc; without microreticulation; lateral margins finely bordered, scarcely rounded. Elytron quite regularly, moderately densely and fairly strongly punctured, with traces of two longitudinal rows of punctures; highly polished and shining; without microreticulation; with a distinct impression on lateral side in anterior third (Fig. 38). Ventral surface: metacoxae, metasternum and abdomen moderately strongly but very sparsely punctured; without microreticulation.

**Male.** Median lobe of aedeagus in ventral aspect slightly tapered from base to apex, expanded before apex, apex truncate (Fig. 14A); in lateral aspect straight, tapered in apical ninth, apex bent downwards (Fig. 14B). Parameres narrow triangular in lateral aspect (Fig. 14C, D).

**Female.** Without secondary sexual characters. Sclerotized spermatheca not found.

##### Variation.

Variation of markings is shown in Fig. 9.

##### Etymology.

This species is dedicated to Maximilian Bramböck, Vienna, Austria. The species epithet is a name in the genitive singular.

##### Habitat.

The species was collected in Yunnan in a 1–2-m wide stream flowing through dense primary forest (Jäch and Ji 2003).

##### Distribution.

Laos: Louang Namtha Province; China: Yunnan Province.

#### ﻿ Microdytes sekaensis

Taxon classificationAnimaliaColeopteraDytiscidae

Okada & Wewalka sp. nov.

1D47CF0C-5B88-54CD-AE37-D1797CB021E7

https://zoobank.org/FC40E421-E223-493B-A4C8-9F9B94978DE2

Figs 5 , 10 , 15

##### Type locality.

Thailand, Bueng Kan Province, Seka District, Ban Tong, 18°08'16"N, 103°59'35"E, alt. 190 m.

##### Type material.

***Holotype*** (THNHM): ♂, “THAI: Bueng Kan Seka Dist., Ban Tong St. 206 (alt. 190 m) 28.XII.2020 R. Okada leg. \ HOLOTYPE Microdytessekaensis sp. nov. Okada & Wewalka 2022” [printed red label]. ***Paratypes*** (37 exs.): 1♂, with same data as the holotype (CGW); 36 exs., Laos, Bolikhamsay Province, Nam Kading NPA, Tad Paloy campsite, 18°21–23'N, 104°09'E, alt. 250–400 m, 24.–28.V.2011, NHMB Basel Laos 2011 Expedition, M. Geiser, D. Hauk, A. Phantala & E. Vongphachan leg. (CGW, CRO, NMB, NMP). All paratypes are provided with printed red paratype labels.

##### Diagnosis.

*Microdytessekaensis* sp. nov. resembles *M.hainanensis* Wewalka, 1997 and *M.schwendingeri* Wewalka, 1997 (Figs 34, 40B) in habitus and size but differs by elytral markings and clypeus not bordered. Size and coloration of *M.sekaensis* sp. nov. is also similar to those of *M.gabrielae* Wewalka, 1997 (Fig. 23), but it is distinguishable from the latter by the more rounded-oval body and finely and sparsely punctured ventral surface.

##### Description.

***Measurements*.**TL = 1.34–1.36 mm (1.36 mm), TL-h = 0.92–0.93 mm (0.93 mm), MW = 0.99–1.01 mm (1.01 mm), TL/MW = 1.35–1.36 (1.35). Body broadly oval, strongly convex (Fig. 5).

***Coloration*.** Head yellowish brown. Pronotum yellowish brown, along posterior margin narrowly dark brown. Elytron reddish brown to dark brown with two yellowish brown spots: one basal spot not reaching base, one anterior lateral spot continuing along the lateral margin to a post-median lateral spot and a transverse spot near apex, also with a round median spot near suture (Figs 5, 10). Ventral surface predominantly yellowish brown. Legs, antennae and palpi yellowish brown.

***Sculpture and structure*.** Head very finely and sparsely, slightly irregularly punctured; entirely and distinctly microreticulate; clypeus not bordered. Pronotum quite irregularly, finely, and sparsely punctured on the disc, additionally with coarser punctures at medial part of posterior margin; without microreticulation; lateral margins finely bordered, regularly rounded. Elytron fairly finely, regularly, and moderately densely punctured, progressively finer and sparser towards lateral margin; two longitudinal rows of punctures moderately distinct; highly polished and shining; without microreticulation. Ventral surface: metacoxae and metasternum finely and sparsely punctured, epipleura and abdomen almost without punctures; without microreticulation.

**Figures 15, 16.**
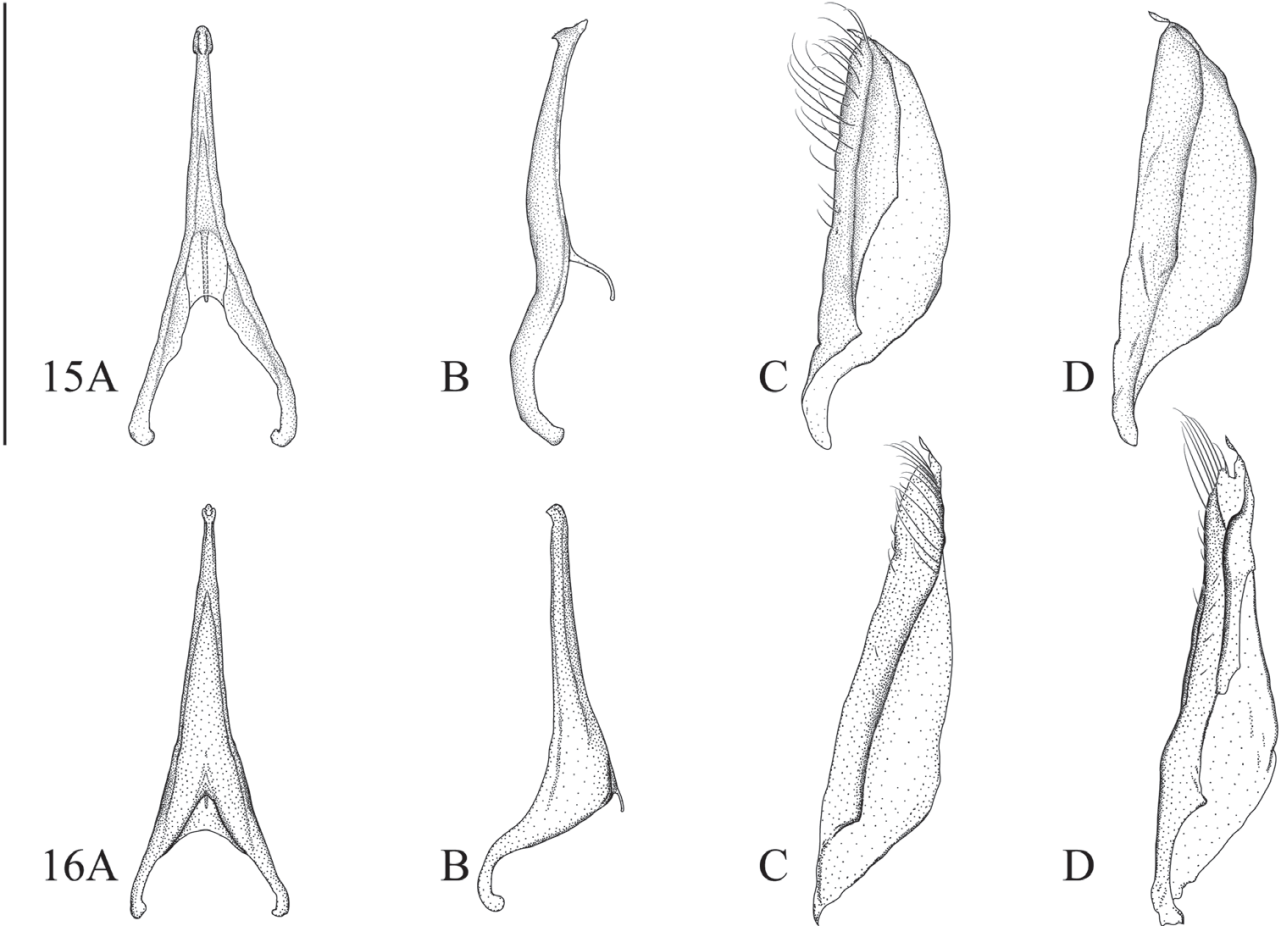
**15***Microdytessekaensis* sp. nov. **16***M.ubonensis* sp. nov. **A** median lobe in ventral aspect **B** median lobe in lateral aspect **C** left paramere in lateral aspect **D** right paramere in medial aspect. Scale bar: 0.25 mm.

**Male.** Median lobe of aedeagus in ventral aspect slightly tapered from base to apex and narrowly constricted before apex, apex spoon shaped (Fig. 15A); in lateral aspect slightly curved with an arrowhead shaped apical expansion (Fig. 15B). Paramere narrowly trapezoid in lateral aspect (Fig. 15C, D).

**Female.** Without secondary sexual characters. Sclerotized spermatheca not found.

##### Variation.

Variation of markings is shown in Fig. 10.

##### Etymology.

This species is named after the district name of the type locality.

##### Habitat.

This species was collected in the remaining water pools in a dried-up stream which had a sandy bottom with rich leaf deposits (Fig. 47).

##### Distribution.

Thailand: Bueng Kan Province; Laos: Bolikhamsay Province.

#### ﻿ Microdytes ubonensis

Taxon classificationAnimaliaColeopteraDytiscidae

Okada & Wewalka sp. nov.

E631B55B-5C05-5D5E-BBEF-1A3FD015833B

https://zoobank.org/007C27AC-BD23-48EE-9659-05448237A6EE

Figs 6 , 11 , 16 , 40A

##### Type locality.

Thailand, Ubon Ratchathani Province, Si Mueang Mai Dist., Nam Thaeng, 15°32'19"N, 105°24'08"E, alt. 200 m.

##### Type material.

***Holotype*** (THNHM): ♂, “THAI: Ubon Ratchathani Si Mueang Mai Dist., Nam Thaeng St. 242 (alt. 200 m) 23.V.2021 R. Okada leg.” [printed white label] / HOLOTYPE Microdytesubonensis sp. nov. Okada & Wewalka 2022” [printed red label]. ***Paratypes*** (52 exs.): 3♂♂, 1♀, with same data as the holotype (CGW, CRO); 4♀♀, Thailand, Ubon Ratchatani Province, Nam Yuen Dist., Dom Pradit St. 221 (alt. 190 m), 14.402°N, 104.215°E, 26.II.2021 R Okada leg. (THNHM); 4♂♂, 4♀♀, Thailand, Ubon Ratchathani Province, Nam Yuen District, Dom Pradit St. 240 (alt. 260 m), 14°23'22"N, 105°09'25"E, 22.V.2021, R. Okada leg. (CRO); 18 exs., Laos, Savannakhet Province, Phou Xang He NBCA, ca. 5 km SW Ban Pa Phaknau, 17°00'N, 105°38'E, alt. 250–400 m, 31.V.–6.VI. 2011, NHMB Basel, Laos 2011 Expedition, M. Brancucci, M. Geiser, D. Hauk, Z. Kraus, A. Phantala & E. Vongphachan leg. (CGW, NMB, NMP); 1♂, Laos, Champasak Province, Bolavens Plateau, waterfall ca. 2 km E Tad Katamtok, 15°08.1'N, 106°38.8'E, alt. 415 m, 10.–12.V.2010, J. Hájek leg. (NMP); 15 exs., Laos, Champasak Province, Bolavens Plateau, ca. 1 km S Ban Lak 40 [vill.] Tad Yueang waterfall, 15°10.8'N, 106°08.3'E, alt. 900–970 m, 28.IV.2010, J. Hájek leg. (CGW, CRO, NMP); 1♂, Laos, Vientiane Province, Phou Khao Khouay NBCA ca. 46 km N Vientiane (waterfall), 18°22.4'N, 102°42.4'E, alt. 270 m, 18.V.2010, J. Hájek leg. (NMP); 1♀, Laos, Vientiane Province, 90 km E Vientiane, Phou Khao Khouay NP, Nam Leuk, 1.–8.VI.1996, H. Schillhammer leg (NMW). All paratypes are provided with printed red paratype labels.

##### Diagnosis.

*Microdytesubonensis* sp. nov. is similar to *M.boukali* Wewalka, 1997 and *M.lotteae* Wewalka, 1998 in habitus, size, and pronotal coloration but differs from these species by having yellowish head and the elytral markings with distinct post-median spot near suture and finely and sparsely punctured ventral surface. *M.ubonensis* sp. nov. also resembles *M.huangyongensis*Bian et al., 2015 in habitus and coloration but it differs in size and male genitalia.

##### Description.

***Measurements*.**TL = 1.25–1.40 mm (1.37 mm), TL-h = 1.08–1.19 mm (1.18 mm), MW = 0.87–0.95 mm (0.95 mm), TL/MW = 1.42–1.47 (1.44). Body regularly oval, moderately convex (Fig. 6).

***Coloration*.** Head yellowish brown. Pronotum dark brown, indistinctly yellowish brown at lateral sides. Elytron dark brown with yellowish brown markings forming a distinct transverse band at base not reaching suture, with a post-median lateral spot and a triangular spot near apex and often with a distinct longitudinal post-median spot near suture (Figs 6, 11). Ventral surface of head and prothorax yellowish brown; thorax, abdominal ventrites and elytral epipleuron reddish brown to dark brown. Legs, antennae and palpi yellowish brown.

***Sculpture and structure*.** Head finely, sparsely, and relatively regularly punctured; almost entirely microreticulate; clypeus not bordered (Fig. 40A). Pronotum quite regularly, sparsely, and fairly strongly punctured, coarser punctures along the posterior margin; without microreticulation; lateral margins very finely bordered, regularly rounded. Elytron moderately regularly and densely punctured, progressively finer and sparser towards lateral margin; longitudinal rows of stronger punctures not distinct; highly polished and shining; without microreticulation. Ventral surface: metacoxae and metasternum finely and sparsely punctured, abdomen without punctures; without microreticulation.

**Male.** Median lobe of aedeagus in ventral aspect equally tapered from base to apex, with a small swelling at apex (Fig. 16A); in lateral aspect tapered from base to apex, with a small ventral hook at apex (Fig. 16B). Paramere narrow triangular in lateral aspect (Fig. 16C, D).

**Figure 17.**
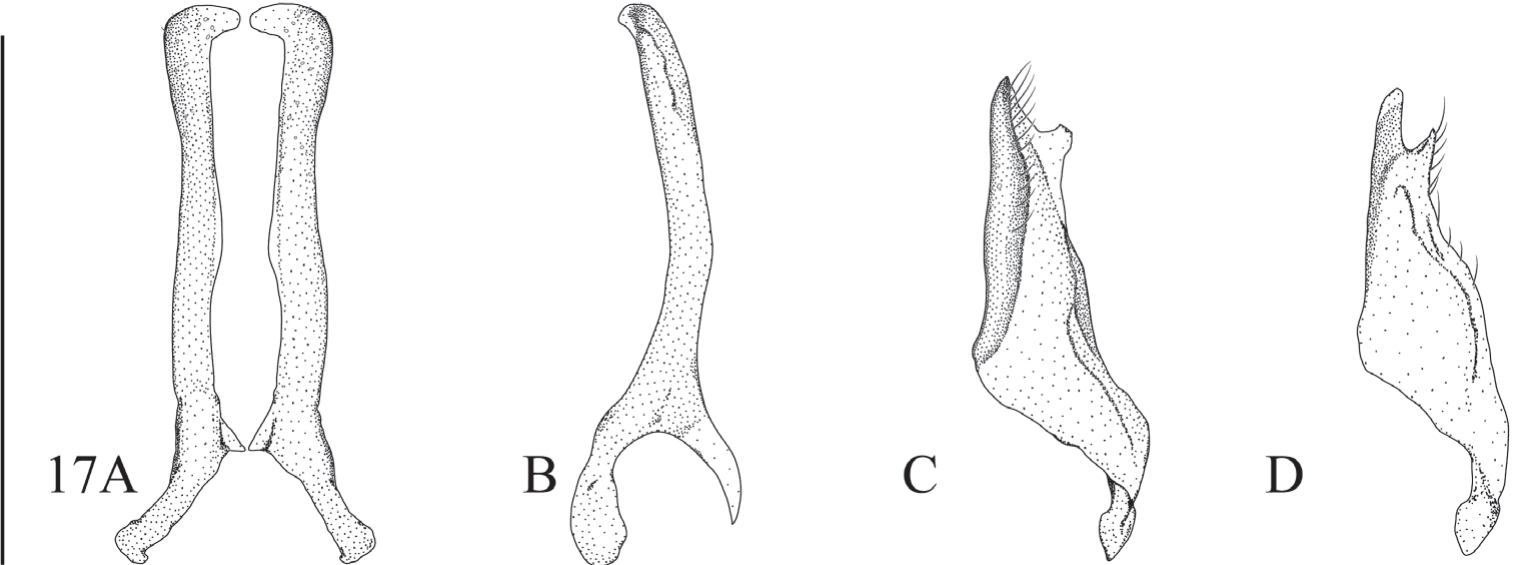
*Microdytesbalkei***A** median lobe in ventral aspect **B** median lobe in lateral aspect **C** left paramere in lateral aspect **D** right paramere in medial aspect. Scale bar: 0.25 mm.

**Female.** Without secondary sexual characters. Sclerotized spermatheca not found.

##### Variation.

Variation of markings is shown in Fig. 11.

##### Etymology.

This species is named after the popular provincial name of the type locality.

##### Habitat.

This species was collected in a variety of habitats. At the type locality, specimens were collected from a small stream on a more or less deforested bedrock (Fig. 48). At other localities, specimens were collected in small pit holes near small forest streams with rich leaf deposits.

##### Distribution.

Thailand: Ubon Ratchathani Province; Laos: Savannakhet, Champasak, and Vientiane provinces.

### ﻿New faunistic records from Thailand, Laos, and Cambodia

#### ﻿ Microdytes akitai

Taxon classificationAnimaliaColeopteraDytiscidae

Wewalka, 1997

34466794-E3B3-5AA9-96FA-CD46E195FF5F

Fig. 18

Microdytes akitai Wewalka, 1997: 17; Wewalka 2011: 37; Nilsson and Hájek 2023: 211.

##### Type locality.

Laos, Vientiane Province, Mt. Phou Khao Khouay.

##### Material examined.

**Figures 18–37.**
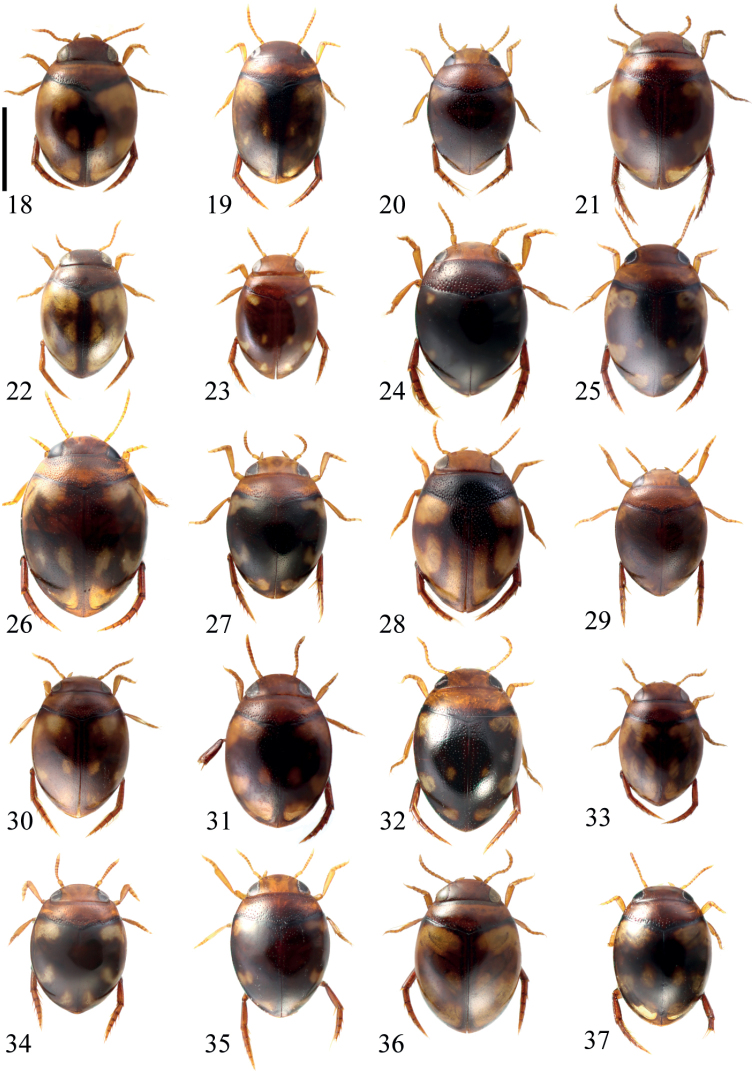
Habitus of **18***Microdytesakitai***19***M.balkei***20***M.dimorphus***21***M.feryi* (paratype, from Myanmar) **22***M.franzi***23***M.gabrielae***24***M.heineri***25***M.maculatus***26***M.mariannae***27***M.menopausis***28***M.paoloi***29***M.pasiricus***30***M.pederzanii***31***M.rocchii***32***M.schoedli***33***M.schoenmanni* (from Myanmar) **34***M.schwendingeri***35***M.shepardi***36***M.shunichii***37***M.zetteli*. Scale bar: 1.0 mm.

Laos: Bolikhamsay Province. 22 exs., Nam Khading National Bio-Diversity Conservation Area, Tad Paloy campsite, 18°23.17'N, 104°09.65'E, alt. 300 m, forest stream, 8.–11.VII.2010 & 24.–28.V.2011, NHMB Basel Laos 2010 & 2011 Expeditions, M. Brancucci, M. Geiser, D. Hauk, A. Phantala & E. Vongphachan leg. (CGW, CRO, NMB, NMP) (Fig. 18).

##### Comments.

Specimens from Bolikhamsay have more distinct elytral markings and darker head color than in the specimens from the type locality but no other differences have been observed, in particular, in the male genitalia.

##### Distribution.

Laos: Vientiane and Bolikhamsay (first record) provinces.

#### ﻿ Microdytes balkei

Taxon classificationAnimaliaColeopteraDytiscidae

Wewalka, 1997

1ADE86AE-29DD-50F2-8720-770561AC9E3B

Figs 17 , 19 , 39D

Microdytes balkei Wewalka, 1997: 18; Wewalka 2011: 29; Nilsson and Hájek 2023: 211.

##### Type locality.

Thailand, Rayong Province, Khao Chamao NP.

##### Material examined.

Thailand: Mukdahan Province. 1♂, Phu Pha Thoep N.P., small pools (23), 1.I.2000, Mazzoldi leg. (CGW); Ubon Ratchathani Province. 1♂, 1♀, Nam Yuen District, Dom Pradit St. 240 (alt. 260 m), 22.V.2021, R. Okada leg. (CRO, THNHM); Saraburi Province. 1♀, Kaeng Khoi District, Ched Khot St. 122 (alt. 140 m), 30.V.2020, R. Okada leg. (CRO); Chonburi Province. 2♂♂, 1♀, Ban Bueng District, Khlong Kiu St. 261 (alt. 200 m), 6.XI.2021, R. Okada leg. (CGW, CRO) (Fig. 17); 2♂♂, 1♀, Si Racha District, Bang Phra St. 269 (alt. 90 m), 26.XII.2021, R. Okada leg. (CRO); Rayong Province. 4♂♂, Wang Chan District, Pa Yup Nai St 299 (alt. 200 m), 17.VII.2022, R. Okada leg. (CRO, THNHM).

Laos: Savannakhet Province. 203 exs., Phou Xang He NBCA, ca. 5 km SW Ban Pa Phaknau, 17°00'N, 105°38'E, alt. 250–400 m, 31.V.–6.VI. 2011, NHMB Basel, Laos 2011 Expedition, M. Brancucci, M. Geiser, D. Hauk, Z. Kraus, A. Phantala & E. Vongphachan leg. (CGW, CRO, NMB, NMP); Sekong Province. 3♀♀, ca. 51 km N Sekong (river) Ho Chi Minh trail, 15°49.6'N, 106°39.8'E, alt. 410 m, 14.–15.V.2010, J. Hájek leg. (NMP).

Cambodia: Koh Hong Province. 12 exs., 20 km SW Koh Hong, Tatai River, 11°34'N, 103°07'E, alt. 50–300 m, 3.–19.V.2005, E. Jendek & O. Šauša leg. (CGW, NMB).

##### Comments.

Since the figure of the median lobe of this species was not depicted clearly in Wewalka (1997), we present a new figure of the aedeagus including parameres (Fig. 17).

##### Distribution.

Thailand: Mukdahan, Rayong, Trat, Ubon Ratchathani (first record), Saraburi (first record), and Chonburi (first record) provinces, Khao Yai NP [Nakhon Ratchatsima or Nakhon Nayok Province]; Laos (first record): Savannakhet and Sekong provinces; Cambodia (first record): Koh Hong Province.

#### ﻿ Microdytes dimorphus

Taxon classificationAnimaliaColeopteraDytiscidae

Wewalka, 1997

0E028AD6-33F1-56FC-96DC-5E7C696DE758

Fig. 20

Microdytes dimorphus Wewalka, 1997: 22; Wewalka 2011: 37; Nilsson and Hájek 2023: 211.

##### Type locality.

Thailand, Nakhon Ratchasima or Nakhon Nayok Province, Khao Yai NP.

##### Material examined.

Thailand: Chonburi Province. 1♂, Si Racha District, Bang Phra St. 269 (alt. 90 m), 26.XII.2020, R. Okada leg. (CRO) (Fig. 20).

##### Comments.

*Microdytesdimorphus* was described based on a single male specimen from Khao Yai National Park and no additional records are known so far. Therefore, this is the second record of this species. Wewalka (1997) suggested *M.dimorphus* is more closely related to *M.menopausis* Wewalka, 1997 (Fig. 27) but differs by having coarser punctures on the elytra similar in size, a punctured abdomen, a more produced clypeus, and larger second segment of antennae. The specimen examined in this study corresponds with *M.dimorphus* due to the status of punctures on the elytra and abdomen, although the characteristics of clypeus and antenna are intermediate between *M.dimorphus* and *M.menopausis*. This species can be distinguished from *M.menopausis* in having a more slender body shape and indistinct elytral markings at both shoulders and post-median part (Fig. 20).

##### Distribution.

Thailand: Khao Yai NP [Nakhon Ratchasima or Nakhon Nayok Province] and Chonburi (first record) Province.

#### ﻿ Microdytes franzi

Taxon classificationAnimaliaColeopteraDytiscidae

Wewalka & Wang, 1998

31E418F3-24E9-5266-9D9F-2B64F15A6BC2

Fig. 22

Microdytes franzi Wewalka & Wang, 1998: 65; Wewalka 2011: 38; Nilsson and Hájek 2023: 211.

##### Type locality.

Laos, Vientiane Province, Mt. Phou Khao Khouay.

##### Material examined.

Laos: Bolikhamsay Province. 20 exs., Nam Khading National Bio-Diversity Conservation Area, Tad Paloy campsite, 18°23.17'N, 104°09.65'E, alt. 300 m, forest stream, 8.–11.VII.2010 & 24.–28.V.2011, NHMB Basel Laos 2010 & 2011 Expeditions, M. Brancucci, M. Geiser, D. Hauk, A. Phantala & E. Vongphachan leg. (CGW, CRO, NMB, NMP) (Fig. 22).

##### Distribution.

Laos: Vientiane and Bolikhamsay (first record) provinces.

#### ﻿ Microdytes gabrielae

Taxon classificationAnimaliaColeopteraDytiscidae

Wewalka, 1997

7F29A223-BC0C-56BE-B0FC-38B35495A93B

Fig. 23

Microdytes gabrielae Wewalka, 1997: 24; Wewalka 2011: 29; Nilsson and Hájek 2023: 211.

##### Type locality.

Thailand, Phetchabun Province, Huai Nam Phang.

##### Material examined.

Thailand: Phetchabun Province. 1♂, Phu Hin Rongkla NP, small stream (7), 25.XII.1999, Mazzoldi leg. (CGW) (Fig. 23); 4♂♂, 3♀♀, Lom Kao District, Ban Noen St. 294 (alt. 1620 m), 18.VI.2022, R. Okada leg. (CGW, CRO, THNHM); Phitsanulok Province. 1♂, Nakhon Thai Distr., Phu Hin Rong Kla NP, in waterfall, 16°59'49.1"N, 101°00'34.8"E, 7.III.2016, A. Damaška leg. (NMP).

##### Distribution.

Thailand: Phetchabun and Phitsanulok provinces.

#### ﻿ Microdytes heineri

Taxon classificationAnimaliaColeopteraDytiscidae

Wewalka, 2011

5BA38C25-DE72-5916-ACB8-998D206BB050

Figs 24 , 39A

Microdytes heineri Wewalka, 2011: 23; Nilsson and Hájek 2023: 211.

##### Type locality.

China, Yunnan Province, Simao Prefecture.

##### Material examined.

Thailand: Chiang Mai Province. 8 exs., Mae Taeng District, Pa Pae St. 49 (alt. 1000 m), 15.VI.2019, R. Okada leg. (CGW, CRO, THNHM); 1♂, same district, Kuet Chang St. 170 (alt. 520 m), 16.VIII.2020, R. Okada leg. (CRO) (Fig. 24).

##### Comments.

Male specimens have distinct setae on the metacoxae (Fig. 39A).

##### Distribution.

Thailand: Nan and Chiang Mai (first record) provinces; Laos: Luang Namtha and Luang Prabang provinces; China: Yunnan Province.

#### ﻿ Microdytes maculatus

Taxon classificationAnimaliaColeopteraDytiscidae

(Motschulsky, 1860)

E88287CB-88D0-56EA-9854-CBECE0AAD8BB

Figs 25 , 39C , 41

Hydrovatus maculatus Motschulsky, 1860: 42; Sharp 1882: 814, 973; Régimbart 1899: 231; Zaitzev 1915: 293; Zimmermann 1920: 34.
Desmopachria maculatus : Gschwendtner 1936: 367; Balfour-Browne 1946: 106 (uncertain assignment).
Microdytes maculatus : Vazirani 1969: 301; Vazirani 1977: 24; Satô 1981: 68; Wewalka 1997: 27; Biström et al. 1997: 75; Miller and Wewalka 2010: 35; Wewalka 2011: 29; Nilsson and Hájek 2023: 211.

##### Type locality.

“Ind or”, “Dohen [a place with this name exists in Pakistan, Jammu and Kashmir Province, but it is very unlikely that this is the type locality]”.

##### Material examined.

Thailand: Chiang Mai Province. 6♂♂, 1♀, Chom Thong District, Ban Luang St. 165 (alt. 360 m), 15.VIII.2020, R. Okada leg. (CGW, CRO) (Fig. 25); 9 exs., Mae Chaem District, Tha Pha St. 166 (alt. 720 m), 15.VIII.2020, R. Okada leg. (CGW, CRO); 1♂, 1♀, Chiang Dao District, Ping Khong St. 226 (alt. 430 m), 20.III.2021, R. Okada leg. (CRO); Chiang Rai Province. 3♀♀, Wiang Kaen District, Muang Yai St. 70 (alt. 470 m), 2.VIII.2019, R. Okada leg. (CRO); Lampang Province. 5♂♂, 9♀♀, Thoen District, Mae Pa St. 197 (alt. 200 m), 15.XI.2020, R. Okada leg. (CGW, CRO); Mae Hong Son Province. 3♂♂, Pai District, Mueang Paeng St. 93 (alt. 760 m), 1.XII.2019, R. Okada leg. (CRO); 3♂♂, 2♀♀, Pha Bong, ca. 12 km S Mae Hong Son, 12.XI.1995, H. Zettel leg (CGW, NMW); Phrae Province. 1♂, 15 km E Phrae, Mae Khaem, 16.XI.1995, H. Zettel leg. (NMW); Uttaradit Province. 2♂♂, 1♀, Nam Pat District, Saen To St. 184 (alt. 210 m), 13.IX.2020, R. Okada leg. (CRO); Phetchabun Province. 1♀, Huai Nam Phang, S Ban Nam Nao, 25.XI.1995, H. Zettel leg. (NMW); Bueng Kan Province. 2♂♂, Seka District, Ban Tong St. 206 (alt. 190 m), 27.XII.2020, R. Okada leg. (CGW, CRO); Nakhon Phanom Province. 12 exs, Bang Phaeng District, Phai Lom St. 203 (alt. 200 m), 27.XII.2020, R. Okada leg. (CGW, CRO); Sakon Nakhon Province. 1♀, Charoen Sin District, Huai Yang St. 208 (alt. 320 m), 29.XII.2020, R. Okada leg. (CRO); 2♂♂, 2♀♀, Tao Ngoi District, Nan Tan St. 210 (alt. 310 m), 29.XII.2020, R. Okada leg. (CGW, CRO); Ubon Ratchathani Province. 1♂, 1♀, Si Mueang Mai District, Nam Thaeng St. 221 (alt. 190 m), 26.II.2021, R. Okada leg. (CRO); 1♂, 1♀, Nam Yuen District, Dom Pradit St. 224 (alt. 190 m), 27.II.2021, R. Okada leg. (CRO); Kanchanaburi Province. 1♂, Si Sawat District, Na Suan St. 28 (alt. 310 m), 12.I.2019, R. Okada leg. (CRO).

**Figures 38–40.**
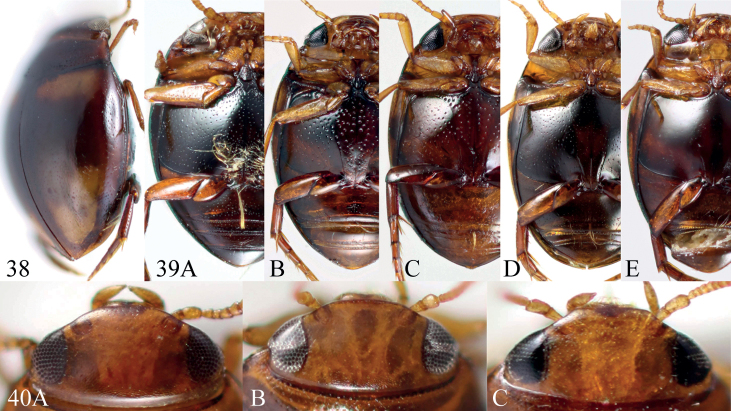
Diagnostic characters of *Microdytes* species. **38** Impression of lateral side (*M.maximiliani* sp. nov.) **39** ventral surface **A** with setae (*M.heineri*) **B** with very coarse punctures (*M.schoedli*) **C** with coarse punctures (*M.maculatus*) **D** with fine punctures (*M.balkei*) **E** with very fine punctures (*M.jeenthongi* sp. nov.) **40** clypeus bead **A** not bordered (*M.ubonensis* sp. nov.) **B** finely bordered (*M.schwendingeri*) **C** produced (*M.dimorphus*).

Laos: Luang Prabang Province. 4 exs., Thong Khan, 19°35'N, 101°58'E, alt. ca. 750 m, 11–12.V.2002, V. Kubáň leg. (CGW, NMB).

##### Comments.

**Figures 41–43.**
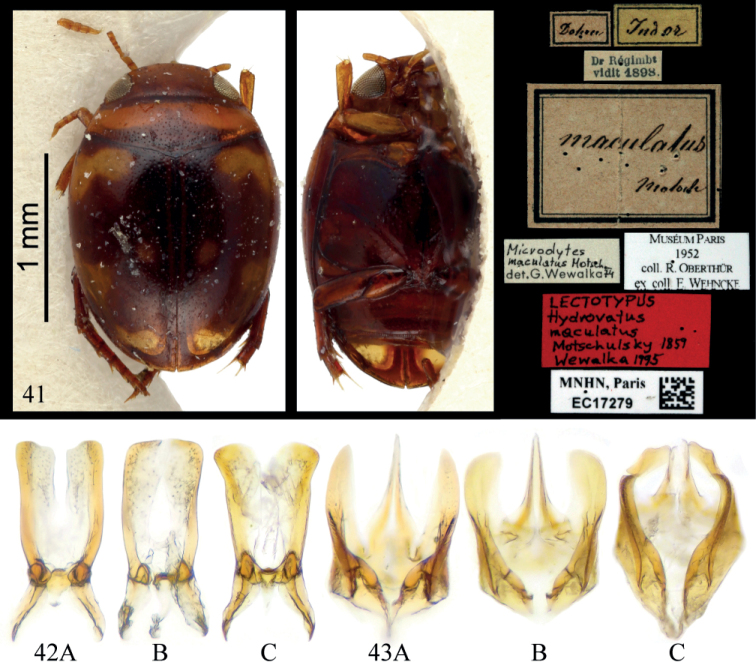
**41** Lectotype of *Microdytesmaculatus* (photograph by Christophe Rivier, Muséum national d’histoire naturelle, Paris) **42** median lobe of **A***M.maculatus* from northern Thailand **B** from northeastern Thailand **C***M.eliasi* sp. nov. **43** paramere of **A***M.maculatus* from northern Thailand **B** from northeastern Thailand **C***M.eliasi* sp. nov.

The lectotype of *Microdytesmaculatus* (Fig. 41) has a rhomboid-like habitus similar to that of most specimens from Myanmar, northern Thailand, and China, while most specimens from northeastern Thailand and Laos have a more regularly rounded habitus, but this is not a discriminant character.

There is also a geographic variation in the shapes of the median lobe and the apical part of the parameres. In specimens from Myanmar, northern Thailand, and China, the two parts of the median lobe have obtuse apical medial angles (Fig. 42A), and the parameres have narrow tips (Fig. 43A), while in those of northeastern Thailand and western Laos the two parts of the median lobe have almost right apical medial angles (Fig. 42B) and the parameres have rounded tips (Fig. 43B). Because some specimens show an intermediate status and no other distinguishing character can be found, they are treated as geographic variations belonging to the same species. Molecular biological studies are required to establish if they are different species within a complex.

##### Distribution.

India: southern Andaman Islands; Myanmar: Chin and Shan states, Saganing Division; Thailand: Chiang Mai, Chiang Rai (first record), Lampang (first record), Lamphun, Mae Hong Son, Uttaradit (first record), Phetchabun (first record), Bueng Kan (first record), Mukdahan, Nakhon Phanom (first record), Sakon Nakhon (first record), Ubon Ratchathani (first record), and Kantchanaburi provinces, Khao Yai NP [Nakhon Ratchasima or Nakhon Nayok Province]; Laos: Vientiane, Luang Prabang (first record) and Khammouan provinces; China: Yunnan Province.

#### ﻿ Microdytes mariannae

Taxon classificationAnimaliaColeopteraDytiscidae

Wewalka, 1997

F6E632D1-B8EB-551B-9F04-3CBB656211DC

Fig. 26

Microdytes mariannae Wewalka, 1997: 28; Wewalka 2011: 30; Nilsson and Hájek 2023: 211.

##### Type locality.

Thailand, Phetchabun Province, Nam Nao.

##### Material examined.

Thailand: Phetchabun Province. 8♂♂, 10♀♀, Lom Kao District, Ban Noen St. 187 (alt. 1620 m), 20.IX.2020, R. Okada leg. (CGW, CRO, THNHM) (Fig. 26); 2♂♂, 2♀♀, same locality, St. 294, 18.VI.2022, R. Okada Leg. (CRO, THNHM).

##### Distribution.

Thailand: Phetchabun and Loei provinces.

#### ﻿ Microdytes menopausis

Taxon classificationAnimaliaColeopteraDytiscidae

Wewalka, 1997

ABD36E1B-AF39-5CFE-9DCC-61C1A970DD2C

Fig. 27

Microdytes menopausis Wewalka, 1997: 29; Wewalka 2011: 30; Nilsson and Hájek 2023: 211.

##### Type locality.

Thailand, Khao Yai NP.

##### Material examined.

Thailand: Nakhon Phanom Province. 1♂, 2♀♀, Bang Phaeng District, Phai Lom St. 203 (alt. 200 m), 27.XII.2020, R. Okada leg. (CGW, CRO); Sakon Nakhon Province. 1♀, Tao Ngoi District, Nan Tan St. 210 (alt. 310 m), 29.XII.2020, R. Okada leg. (CRO) (Fig. 27); Ubon Ratchathani Province. 1♀, Nam Yuen District, Dom Pradit St. 224 (alt. 190 m), 27.II.2021, R. Okada leg. (CGW); 1♂, Si Mueang Mai District, Nam Thaeng St. 241 (alt. 190 m), 23.V.2021, R. Okada leg. (THNHM).

Laos: Khammouan Province. 1♂, Nakai-Nam Theun NPA, Ban Navang env., 17°57–59'N, 105°13–16'E, alt. 600–750 m, 18.–21.V.2012, NHMB Basel, Laos 2012 Expedition, M. Brancucci, M. Geiser, K. Phanthavong & S. Xayalath leg. (NMB); Savannakhet Province. 3 exs., Phou Xang He NBCA, ca. 5 km SW Ban Pa Phaknau, 17°00'N, 105°38'E, alt. 250–400 m, 31.V.–6.VI. 2011, NHMB Basel, Laos 2011 Expedition, M. Brancucci, M. Geiser, D. Hauk, Z. Kraus, A. Phantala & E. Vongphachan leg. (CGW, NMB).

##### Distribution.

Thailand: Trat, Nakhon Phanom (first record), Sakon Nakhon (first record), and Ubon Ratchathani (first record) provinces, Khao Yai NP [Nakhon Ratchasima or Nakhon Nayok Province]; Laos: Khammouan, Savannakhet (first record) and Sekong provinces.

#### ﻿ Microdytes paoloi

Taxon classificationAnimaliaColeopteraDytiscidae

Wewalka, 2011

A442A162-3EAA-5C26-9C5E-43F6FE525BFC

Fig. 28

Microdytes paoloi Wewalka, 2011: 24; Nilsson and Hájek 2023: 211.

##### Type locality.

Thailand, Phetchabun Province, Phu Hin Rongkla NP.

##### Material examined.

Thailand: Phetchabun Province. 1♂, paratypus, Phu Hin Rongkla NP, small stream, alt. 1250 m (4), 25.XII.1999, Mazzoldi leg. (CMW) (Fig. 28).

##### Distribution.

Thailand: Phetchabun and Loei provinces.

#### ﻿ Microdytes pasiricus

Taxon classificationAnimaliaColeopteraDytiscidae

(Csiki, 1938)

556A83E1-8500-53D8-8122-6F8B74C93A32

Fig. 29

Hydrovatus pasiricus Csiki, 1938: 126 (including var. simplicor and var. unicolor).
Microdytes pasiricus : Biström et al. 1997: 75; Wewalka 1997: 30; Wewalka 2011: 30; Freitag et. al. 2016: 185; Nilsson and Hájek 2023: 212.

##### Type locality.

Indonesia, Central Java, Sarangan near Lake Pasir.

##### Material examined.

Thailand: Chiang Rai Province. 3♂♂, Wiang Kaen District, Muang Yai St. 71 (alt. 440 m), 11.VIII.2019, R. Okada leg. (CGW, CRO) (Fig. 29); Chiang Mai Province. 2♀♀, Mae Chaem District, Tha Pha St. 166 (alt. 720 m), 15.VIII.2020, R. Okada leg. (CRO, THNHM).

##### Distribution.

Thailand: Chiang Rai (first record), Chiang Mai (first record) and Phetchabun provinces; Philippines: Busuanga, Luzon, Mindoro, Palawan; Singapore; Indonesia: Java.

#### ﻿ Microdytes pederzanii

Taxon classificationAnimaliaColeopteraDytiscidae

Wewalka, 2011

197593CB-CB38-5EBC-BE29-E151501763E4

Fig. 30

Microdytes pederzanii Wewalka, 2011: 25; Nilsson and Hájek 2023: 212.

##### Type locality.

Thailand, Phetchabun Province, Phu Hin Rongkla NP.

##### Material examined.

Thailand: Phetchabun Province. 2♀♀, Lom Kao District, Ban Noen St. 187 (alt. 1620 m), 20.IX.2020, R. Okada leg. (CRO); Uttaradit Province. 1♂, 1♀, Nam Pad District, Phu Soi Dao NP, pine forest, 12.VII.2020, T. Jeenthong leg. (THNHM) (Fig. 30).

##### Distribution.

Thailand: Phetchabun and Uttaradit (first record) provinces.

#### ﻿ Microdytes rocchii

Taxon classificationAnimaliaColeopteraDytiscidae

Wewalka, 2011

0A4BB3A4-07CE-548D-9DAE-D53A6D2C6516

Fig. 31

Microdytes rocchii Wewalka, 2011: 26; Nilsson and Hájek 2023: 212.

##### Type locality.

Laos, Khammuan Province, Ban Khoun Ngeun.

##### Material examined.

Laos: Khammouan Province. 1♀, Ban Khon Ngeun, 18°07'N, 104°29'E, alt. ca 200 m, 19.–31.V.2001, Pacholátko leg. (CGW) (Fig. 31); Savannakhet Province. 1♂, Phou Xang He NBCA, ca. 5 km SW Ban Pa Phaknau, 17°00'N, 105°38'E, alt. 250–400 m, 31.V.–6.VI. 2011, NHMB Basel, Laos 2011 Expedition, M. Brancucci, M. Geiser, D. Hauk, Z. Kraus, A. Phantala & E. Vongphachan leg. (NMB).

##### Distribution.

Laos: Khammuan and Savannakhet (first record) provinces.

#### ﻿ Microdytes schoedli

Taxon classificationAnimaliaColeopteraDytiscidae

Wewalka, 1997

C58B0410-5D26-57D0-9BCA-3D874C15E5E9

Figs 32 , 39B

Microdytes schoedli Wewalka, 1997: 33; Wewalka 2011: 31; Nilsson and Hájek 2023: 212.

##### Type locality.

Thailand, Phetchabun Province, Nam Nao NP.

##### Material examined.

Thailand: Chiang Mai Province. 2♀♀, Chiang Dao District, Ping Khong St. 226 (alt. 430 m), 20.III.2021, R. Okada leg. (CRO); Lampang Province. 1♂, 1♀, Thoen District, Mae Pa St. 197 (alt. 200 m), 15.XI.2020, R. Okada leg. (CRO); Mae Hong Son Province. 1♂, Pai District, Thung Yao St. 50 (alt. 610 m), 15.VI.2019, R. Okada leg. (CRO); 2♂♂, 2♀♀, Muang Mae Hong Son District, Pha Bong St. 250 (alt. 480 m), 13.VI.2021, R. Okada leg. (CGW, CRO) (Fig. 32); Phetchabun Province. 1♂, 2♀♀, Lom Sak District, Nam Chun St. 296 (alt. 200 m), 19.VI.2022, R. Okada leg. (CRO); Kanchanaburi Province. 2♂♂, 1♀, Si Sawat District, Khao Chot St. 27 (alt. 420 m), 12.I.2019, R. Okada leg. (CRO); 1♀, same district, Na Suan St. 28 (alt. 310 m), 12.I.2019, R. Okada leg. (CRO).

Laos: Khammouan Province. 1♂, 2♀♀, Nakai-NamTheun NPA, Ban Navang env., 17°57–59'N, 105°13–16'E, alt. 600–750 m, 18.–21.V.2012, NHMB Basel, Laos 2012 Expedition, M. Brancucci, M. Geiser, K. Phanthavong & S. Xayalath leg. (NMB); Savannakhet Province. 1♂, Phou Xang He NBCA, ca. 5 km SW Ban Pa Phaknau, 17°00'N, 105°38'E, alt. 250–400 m, 31.V.–6.VI. 2011, NHMB Basel, Laos 2011 Expedition, M. Brancucci, M. Geiser, D. Hauk, Z. Kraus, A. Phantala & E. Vongphachan leg. (NMB).

##### Distribution.

Thailand: Chiang Mai, Lampang (first record), Mae Hong Son (first record), Phetchabun, Tak, Mukdahan, and Kanchanaburi (first record) provinces; Laos: Khammouan (first record), Savannakhet (first record) and Sekong provinces.

#### ﻿ Microdytes schwendingeri

Taxon classificationAnimaliaColeopteraDytiscidae

Wewalka, 1997

B70D2C12-B4DC-50A0-B553-DCC9BD80B24A

Figs 34 , 40B

Microdytes schwendingeri Wewalka, 1997: 36; Wewalka 2011: 37; Nilsson and Hájek 2023: 212.

##### Type locality.

Thailand, Sakon Nakhon Province, Phu Pan NP.

##### Material examined.

Thailand: Nakhon Phanom Province. 1♂, 1♀, Bang Phaeng District, Phai Lom St. 203 (alt. 200 m), 27.XII.2020, R. Okada leg. (CRO, THNHM) (Fig. 34).

Laos: Savannakhet Province, 1♀, Phou Xang He NBCA, ca. 5 km SW Ban Pa Phaknau, 17°00'N, 105°38'E, alt. 250–400 m, 31.V.–6.VI. 2011, NHMB Basel, Laos 2011 Expedition, M. Brancucci, M. Geiser, D. Hauk, Z. Kraus, A. Phantala & E. Vongphachan leg. (NMB); Champasak Province. 2♂♂, 1♀, Bolavens Plateau, waterfall ca. 2 km E Tad Katamtok, 15°08.1'N, 106°38.8'E, alt. 415 m, 10.–12.V.2010, J. Hájek leg. (CGW, NMP).

##### Comments.

Some specimens from Laos have less distinct elytral markings but no other differences from typical specimens have been observed.

##### Distribution.

Thailand: Nakhon Phanom (first record) and Sakon Nakhon provinces; Laos (first record): Savannakhet and Champasak provinces.

#### ﻿ Microdytes shepardi

Taxon classificationAnimaliaColeopteraDytiscidae

Wewalka, 1997

FAAD1C24-D633-582C-AA7D-E3F55B9F8B09

Fig. 35

Microdytes shepardi Wewalka, 1997: 37; Wewalka 2011: 33; Nilsson and Hájek 2023: 212.

##### Type locality.

Thailand, Phetchabun Province, Nam Nao NP.

##### Material examined.

Thailand: Chiang Mai Province. 1♂, 1♀, Chiang Dao District, Ping Khong St. 226 (alt. 430 m), 20.III.2021, R. Okada leg. (CRO); Chiang Rai Province. 1♂, Wiang Kaen District, Muang Yai St. 71 (alt. 440 m), 2.VIII.2019, R. Okada leg. (CRO); Uthai Thani Province. 5♂♂, 2♀♀, Ban Rai District, Kaen Makrut St. 128 (alt. 440 m), 20.VI.2020, R. Okada leg. (CGW, CRO, THNHM) (Fig. 35).

Laos: 1♂, Luang Prabang Province, Thong Khan, 19°35'N, 101°58'E, alt. ca. 750 m, 11.–12.V.2002, V. Kubáň leg. (NMB).

##### Distribution.

Thailand: Chiang Mai, Chiang Rai (first record), Mae Hong Son, Phetchabun, Uthai Thani (first record), Chaiyaphum and Sakon Nakhon provinces; Laos: Luang Prabang Province; China: Yunnan Province.

#### ﻿ Microdytes shunichii

Taxon classificationAnimaliaColeopteraDytiscidae

Satô, 1995

44C6F270-9CAB-5C19-B4AC-75C7B962567D

Fig. 36

Microdytes shunichii Satô, 1995: 313; Wewalka 1997: 38; Wewalka 2011: 33; Nilsson and Hájek 2023: 212.
Microdytes holzmanni Wewalka & Wang, 1998: 66.
Microdytes holzmannorum : Nilsson 2007: 51 (as unjustified emendation of holzmanni).

##### Type locality.

Vietnam, Vinh Phuc Province, Mt. Tam Dao.

##### Material examined.

Thailand: Phetchabun Province. 1♀, Lom Kao District, Ban Noen St. 187 (alt. 1620 m), 20.IX.2020, R. Okada leg. (CRO); 3♂♂, 2♀♀, same locality, St. 294, 18.VI.2022, R. Okada leg. (CGW, CRO, THNHM).

Laos: Luang Namtha Province. 62 exs., 10 km E Muang Sing, Ban Oudomsinh / B. Nam Det / B. Nam Mai, 21°09–10'N, 101°13–15'E, 750–1400 m, 14.–20.V.2011, NHMB Basel, Laos 2011 Expedition, D. Hauk & M. Geiser (CGW, CRO, NMB, NMP) (Fig. 36); Luang Prabang Province. 1♂, 5 km W Ban Song Cha, 20°33–34'N, 102°14'E, 1200 m, 24.–30. IV.1999, V. Kubáň leg. (NMB); Khammouan Province. 2♂♂, Nakai-Nam Theun NPA, Ban Navang env., 17°57–59'N, 105°13–16'E, alt. 600–750 m, 18.–21.V.2012, NHMB Basel, Laos 2012 Expedition, M. Brancucci, M. Geiser, K. Phanthavong & S. Xayalath leg. (NMB); Savannakhet Province. 1♀, Phou Xang He NBCA, ca. 5 km SW Ban Pa Phaknau, 17°00'N, 105°38'E, alt. 250–400 m, 31.V. – 6.VI. 2011, NHMB Basel, Laos 2011 Expedition, M. Brancucci, M. Geiser, D. Hauk, Z. Kraus, A. Phantala & E. Vongphachan leg. (CGW, NMB, NMP); Attapeu Province. 1♂, 1♀, Dong Amphan National Bio-Diversity Conservation Area, Nong Fa (crater lake) env., 15°05.9'N, 107°25.6'E, alt. ca. 1160 m, 30.IV.–6.V.2010, J. Hájek leg. (NMP); 1♀, Thong Kai Ohk, Ban Kachung (Mai) env., 15°01–02'N, 107°26–27'E, 1200–1450 m, 10.–24.VI.2011, NHMB Basel Laos 2011 Expedition, M. Geiser, D. Hauk, A. Phantala & E. Vongphachan leg. (NMB).

##### Distribution.

Thailand: Nan, Chiang Mai, and Phetchabun (first record) provinces; Laos: Phongsali (first record), Luang Namtha (first record), Oudomxai, Luang Prabang (first record), Vientiane, Khammouan, Savannakhet (first record), and Attapeu (first record) provinces; China: Yunnan Province, Hong Kong; Vietnam: Vinh Phuc Province.

#### ﻿ Microdytes wewalkai

Taxon classificationAnimaliaColeopteraDytiscidae

Bian & Ji, 2009

4A95238C-B149-5BED-9839-DA08716D810B

Microdytes wewalkai Bian & Ji, 2009: 37; Wewalka 2011: 36; Nilsson and Hájek 2023: 212.

##### Type locality.

China, Hainan, Lingtou (Ledong) County, Jianfengling NR.

##### Material examined.

Laos: Attapeu Province. 3♂♂, 2♀♀, Dong Amphan National Bio-Diversity Conservation Area, Nong Fa (crater lake) env., 15°05.9'N, 107°25.6'E, alt. ca. 1160 m, 30.IV.–6.V.2010, J. Hájek leg. (CGW, NMP).

##### Comments.

Specimens from Laos have two transverse yellowish red markings on both sides of the middle of pronotum while in specimens from China (Hainan) it is completely black, but no other differences have been observed.

##### Distribution.

Laos (first record): Attapeu Province; China: Hainan Province.

#### ﻿ Microdytes zetteli

Taxon classificationAnimaliaColeopteraDytiscidae

Wewalka, 1997

67258B2E-E533-5F90-B5BA-B80FA058D96B

Fig. 37

Microdytes zetteli Wewalka, 1997: 41; Wewalka 2011: 34; Nilsson and Hájek 2023: 212.

##### Type locality.

Thailand, Chiang Mai Province, Doi Suthep.

##### Material examined.

Thailand: Chiang Mai Province. 12 exs., Mae Taeng District, Pa Pae St. 49 (alt. 1000 m), 15.VI.2019, R. Okada leg. (CGW, CRO, THNHM); 2♂♂, Mae Chaem District, Tha Pha St. 136 (alt. 720 m), 4.VII.2020, R. Okada leg. (CGW, CRO); 1♂, same locality St. 166 (alt. 720 m), 15.VIII.2020, R. Okada leg. (THNHM) (Fig. 37); Chiang Rai Province. 1♀, Wiang Kaen District, Muang Yai St. 71 (alt. 440 m), 2.VIII.2019, R. Okada leg. (CRO).

Laos: 4 exs., Luang Prabang Province, 5 km W Ban Song Cha, 20°33–4'N, 102°14'E, alt. ca. 1200 m, 1.–16.V.1999, V. Kubáň leg. (CGW, NMB).

##### Distribution.

Myanmar: Shan State; Thailand: Chiang Mai, Chiang Rai (first record) and Mae Hong Son provinces; Laos: Luang Prabang Province.

###### ﻿Additional species known from Thailand, Laos, or Cambodia

#### ﻿ Microdytes schoenmanni

Taxon classificationAnimaliaColeopteraDytiscidae

Wewalka, 1997

E1914898-99C2-5EDA-81A2-D993849DADE4

Fig. 33

Microdytes schoenmanni Wewalka, 1997: 34; Miller and Wewalka 2010: 35; Wewalka 2011: 31; Nilsson and Hájek 2023: 212.

##### Type locality.

Thailand, Trat Province, Koh Chang Island, Klong Prao.

##### Material examined.

Myanmar: 1♂, Shan State, N Aungban, halfway between Pindaya and Ye’ngan, 20°58.271'N, 96°32.488'E, ca. alt. 1241 m, stream, 10.VI.2004, Shaverdo leg. (CGW) (Fig. 33).

##### Distribution.

India: Darjeeling; Nepal; Myanmar: Chin and Shan states, Saganing and Tanintharyi divisions; Thailand: Trat Province, Khao Yai NP [Nakhon Ratchasima or Nakhon Nayok Province]; Laos: Attapeu Province; China: Yunnan Province.

### ﻿Checklist of the species of *Microdytes* from Thailand, Laos, and Cambodia

*Microdytesakitai* Wewalka, 1997 Laos

*Microdytesbalkei* Wewalka, 1997 Thailand, Laos, Cambodia

*Microdytesdimorphus* Wewalka, 1997 Thailand

*Microdyteseliasi* Wewalka & Okada, sp. nov. Thailand, Cambodia

*Microdytesfranzi* Wewalka & Wang, 1998 Laos

*Microdytesgabrielae* Wewalka, 1997 Thailand

*Microdytesheineri* Wewalka, 2011 Thailand, Laos, China

*Microdytesjeenthongi* Okada & Wewalka, sp. nov. Thailand

*Microdytesmaculatus* (Motschulsky, 1860) India, Myanmar, Thailand, Laos, China

*Microdytesmariannae* Wewalka, 1997 Thailand

*Microdytesmaximiliani* Wewalka & Okada, sp. nov. Laos, China

*Microdytesmenopausis* Wewalka, 1997 Thailand, Laos

*Microdytespaoloi* Wewalka, 2011 Thailand

*Microdytespasiricus* (Csiki, 1938) Thailand, Philippines, Singapore, Indonesia

*Microdytespederzanii* Wewalka, 2011 Thailand

*Microdytesrocchii* Wewalka, 2011 Laos

*Microdytesschoedli* Wewalka, 1997 Thailand, Laos

*Microdytesschoenmanni* Wewalka, 1997 India, Nepal, Myanmar, Thailand, Laos, China

*Microdytesschwendingeri* Wewalka, 1997 Thailand, Laos

*Microdytessekaensis* Okada & Wewalka, sp. nov. Thailand, Laos

*Microdytesshepardi* Wewalka, 1997 Thailand, Laos, China

*Microdytesshunichii* Satô, 1995 Thailand, Laos, China, Vietnam

*Microdytesubonensis* Okada & Wewalka, sp. nov. Thailand, Laos

*Microdyteswewalkai* Bian & Ji, 2009 Laos, China

*Microdyteszetteli* Wewalka, 1997 Myanmar, Thailand, Laos

### ﻿Key to the species of *Microdytes* from Thailand, Laos, and Cambodia

**Table:** 

1	Elytron with a distinct impression on lateral side in anterior third (Fig. 38); TL: 1.60–2.00 mm	***M.maximiliani* sp. nov.**
–	Elytron without impression on lateral side	**2**
2	Punctures on metacoxae very coarse (Fig. 39B) to moderately coarse (Fig. 39C)	**3**
–	Punctures on metacoxae fine (Fig. 39D) or very fine (Fig. 39E)	**18**
3	Elytra with two postmedian longitudinal bands (Fig. 28); TL: 1.90–2.00 mm	***M.paoloi***
–	Elytra without longitudinal band	**4**
4	Pronotum dark brown to black with a transverse reddish-brown band on each side. TL: 1.90–2.10 mm	***M.wewalkai***
–	Pronotum without transverse band	**5**
5	Body oval or rhomboid (Figs 20, 24, 27, 29, 35); medium to large species TL: 1.40–2.10 mm	**6**
–	Body oval; small species TL: 1.30–1.40 mm	**11**
6	Elytra without a post-median spot near suture	**7**
–	Elytra with a post-median spot near suture	**13**
7	Pronotum predominantly reddish brown to dark brown; metacoxae of male with distinct setae (Fig. 39A); TL: 1.85–1.95 mm	***M.heineri***
–	Pronotum predominantly yellowish brown; metacoxae without setae	**8**
8	Body regularly oval; cypeus not bordered; TL: 1.70–2.00 mm	***M.shepardi***
–	Body rhomboid; clypeus bordered at least in the middle	**9**
9	Elytra predominantly yellowish brown; clypeus rounded and finely bordered; TL: 1.40–1.60 mm	***M.pasiricus***
–	Elytra predominantly dark brown; clypeus straightened and slightly bordered in the middle (Fig. 40C)	**10**
10	Apical three sternites distinctly punctured; the coarser punctures on the elytra similar in size; TL: 1.75–1.85 mm	***M.dimorphus***
–	Apical three sternites almost without punctures; the coarser punctures on the elytra distinctly of two kinds; TL: 1.40–1.70 mm	***M.menopausis***
11	Body oblong oval; pronotum predominantly dark brown; TL: 1.25–1.40 mm	***M.ubonensis* sp. nov.**
–	Body broadly oval; pronotum predominantly yellowish brown	**12**
12	Elytral markings with distinct transversal band at the base and without a post-median spot near the suture; TL: 1.30–1.40 mm	***M.schwendingeri***
–	Elytral markings with two indistinct spots at the base and with a post-median spot near the suture; TL: 1.34–1.36 mm	***M.sekaensis* sp. nov.**
13	Elytral punctures consisting of one kind	**14**
–	Elytral punctures consisting of two kinds (e.g., Fig. 32)	**15**
14	Body regularly oval; the two parts of the median lobe obtuse to right apical medial angles but not expanded laterally at apex; the tips of paramere narrow to rounded but not constricted; TL: 1.60–1.90 mm	***M.maculatus***
–	Body more regularly oval; the two parts of the median lobe expanded laterally at apex; the tips of paramere constricted; TL: 1.64–1.85 mm	***M.eliasi* sp. nov.**
15	Body oblong oval; TL: 1.90–2.15 mm	***M.schoedli***
–	Body regularly oval	
16	Elytral marking near the base indistinct; bigger punctures on elytra coarser; TL: 1.60–1.75 mm	***M.pederzanii***
–	Elytral marking near the base distinct; bigger punctures on elytra less coarse	**17**
17	Post-median spot near the suture rounded; TL: 1.80–1.85 mm	***M.rocchii***
–	Post-median spot near the suture longitudinal; TL: 2.10–2.25 mm	***M.mariannae***
18	Body oblong oval; TL: 1.70–1.80 mm	***M.balkei***
–	Body regularly oval	**19**
19	Elytral marking near the base indistinct and small spot; TL: 1.45–1.55	***M.gabrielae***
–	Elytral marking near the base distinct	**20**
20	Elytral marking dilated or waved at the shoulder; TL: 1.40–1.65 mm	**21**
–	Elytral marking broad transverse band at the shoulder; TL: 1.60–1.90 mm	**22**
21	Head dark brown; pronotum dark brown; TL: 1.40–1.60 mm	***M.franzi***
–	Head yellowish brown; pronotum reddish brown; 1.40–1.65 mm	***M.schoenmanni***
22	Pronotal punctures coarser; elytral punctures stronger; TL: 1.60–1.70 mm	***M.akitai***
–	Pronotal punctures spacer; elytral punctures finer	**23**
23	Pronotum predominantly yellowish brown; elytral punctures fine but distinct; TL: 1.65–1.90 mm	***M.shunichii***
–	Pronotum predominantly reddish brown; elytral punctures very fine and very sparse	**24**
24	Head moderately finely and sparsely punctured; TL: 1.55–1.70 mm	***M.zetteli***
–	Head finely and sparsely punctured; TL: 1.79–1.82 mm	***M.jeenthongi* sp. nov.**

## ﻿Discussion

### ﻿Distribution patterns

**Figure 44.**
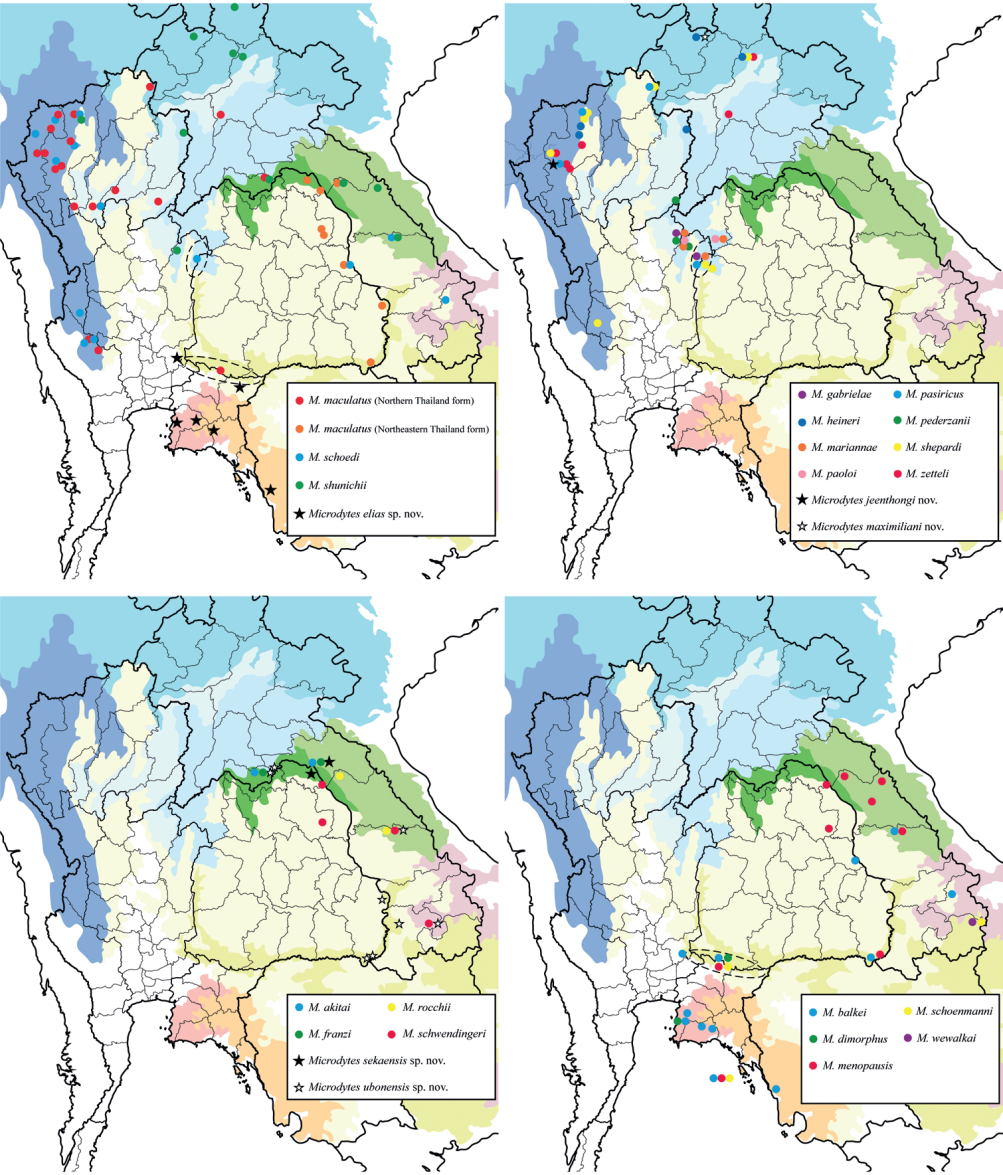
Distribution map of *Microdytes* species in Thailand, Laos, and Cambodia.

The discovery of five new species with three first country records and 40 first regional records from Thailand, Laos, and Cambodia shows how poorly the Dytiscidae fauna of these countries is known. The data on species diversity of *Microdytes* species are summarized in Table 1 and Fig. 44. A total of 25 *Microdytes* species were recorded from these three countries. Among the recorded species, 20 are known from Thailand, 17 from Laos, and two from Cambodia; 16 species (64%) are known to occur only from those three countries, whereas nine species (36%) are also recorded in adjacent countries. This result makes Thailand and Laos the most and second-most fauna-rich countries for this genus.

**Table 1.** Summary of distribution types of *Microdytes* species in main ecoregions in Thailand, Laos, and Cambodia. **T** Thailand **L** Laos **C** Cambodia **KAY** Kayah-Karen montane rain forest **NIN** northern Indochina subtropical forests **LUA** Luang Prabang montane rain forests **NAN** northern Annamites rain forests **NKH** northern Khorat Plateau moist deciduous forests **SEI** southeastern Indochina dry evergreen forests **CIN** central Indochina dry forests **CAR** Cardamom Mountains rain forests **CHA** Chao Phraya lowland moist deciduous forests.

Distribution pattern	*Microdytes* species	Distribution records			Limited to	Main ecoregions in Thailand, Laos, and Cambodia									N	Ratio
		Thailand	Laos	Cambodia	three countries	KAY	NIN	LUA	NAN	NKH	SEI	CIN	CAR	CHA		
Widespread	*maculatus*	+	+			T	T	T/L	L	T	T	T			5	20%
	*pasiricus*	+				T	T	T								
	*schoedli*	+	+		+	T	T	T/L	L			T/L				
	*schuichii*	+	+			T	L	L	L	L	L					
	*schoenmanni*	+	+								T	L	T			
N Thailand and Laos	*heineri*	+	+			T	T/L								9	36%
	*jeenthongi* sp. nov.	+			+	T										
	*maximiliani* sp. nov.		+				L									
	*shepardi*	+	+			T	T	T/L								
	*zetteli*	+	+			T	T	L								
(limited to N Thailand)	*gabrielae*	+			+			T								
	*mariannae*	+			+			T								
	*paoloi*	+			+			T								
	*pederzanii*	+			+			T								
NE Thailand to S Laos	*akitai*		+		+			L	L						6	24%
	*franzi*		+		+			L	L							
	*rocchii*		+		+				L							
	*schwendingeri*	+	+		+					T	L	T				
	*sekaensis* sp. nov.	+	+		+				L	T						
	*ubonensis* sp. nov.	+	+		+			L	L	L	T					
NE and E Thailand to S Laos	*balkei*	+	+	+	+				L		T	L	T/C	T	5	20%
	*dimorphus*	+			+						T			T		
	*eliasi* sp. nov.	+		+	+							T	T/C	T		
	*menopausis*	+	+		+				L	T	T	T	T			
	*wewalkai*		+									L				
Total recorded species		20	17	2	16	8	8	13	10	6	8	8	4	3	25	100%
Ratio					64%	32%	32%	52%	40%	24%	32%	32%	16%	12%		

**Notes.** Ecoregions where only 1 or 2 *Microdytes* species were recorded are excluded in the list (i.e., northern Thailand-Laos moist deciduous forests, southern Annamites montane rain forests, and Chao Phraya lowland moist deciduous forests).

In view of species diversity by ecoregions, LUA has the highest number of species (13 species, 52% of total fauna). This region comprises four species which occur only in one ecoregion. NAN has the second highest number of species (10 species, 40%), including the two new species *M.sekaensis* sp. nov. and *M.ubonensis* sp. nov. KAY, NIN, SEI, and CIN also have high numbers of species (8 species, 32%), including *M.jeenthongi* sp. nov., *M.maximiliani* sp. nov., *M.ubonensis* sp. nov., and *M.eliasi* sp. nov., respectively.

The distribution pattern of *Microdytes* species recorded from the three countries represents four types: 1) widespread type, occurring throughout continental Southeast Asia to adjacent countries (5 species, 20%); 2) northern type, distributed mainly from northern Thailand and Laos to adjacent countries (9 species, 36%); 3) central type, recorded only from northeastern Thailand to southern Laos (6 species, 24%); 4) eastern type, occurring in northeastern and eastern Thailand and southern Laos (5 species, 20%). The conclusions made above are not definitive because there are still many unexplored areas.

Most of the *Microdytes* specimens examined in this study were collected at lotic habitats associated with running water flowing under primary or secondary forests, and many species were sympatric. At one locality of Tha Pha, northern Thailand, located at Kayah-Karen montane rain forests, four species were collected from the same small stream (Fig. 46). In this stream, *M.maculatus*, which shows a wide distribution, was found at several sites: at the edge of a water body, among roots of trees in the gaps between stone and gravel. The other species, however, were collected only in restricted areas: *M.jeenthongi* sp. nov. from a shallow, relatively fast-flowing area where tree roots were exposed at bottom of the river, *M.zetteli* from calm pool areas associated with slowly running water, and *M.pasiricus* among gravel under big stones in fast streams. Although our field surveys were conducted only two times at this site, it is worth noting that *M.jeenthongi* sp. nov. and *M.zetteli*, which morphologically resemble each other, were collected each time at the same places which are only one meter from each other. This result suggests that microhabitat preferences for some *Microdytes* species are very strict, and it may lead to the discovery of the richest fauna in this area.

**Figures 45–48.**
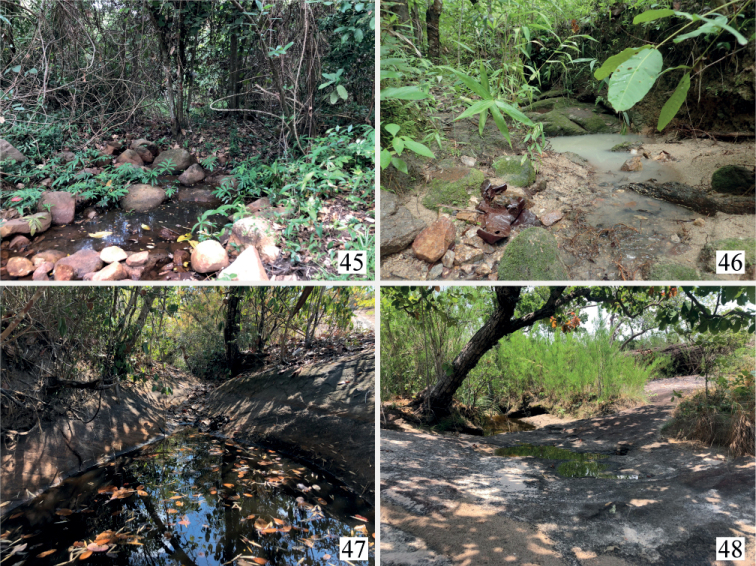
Collecting localities of *Microdytes* species in Thailand **45** Ban Kaeng, Sa Kaeo Province, one of the localities of *M.eliasi* sp. nov. **46** Tha Pha, Chiang Mai Province, type locality of *M.jeenthongi* sp. nov. **47** Ban Tong, Bueng Kan Province, type locality of *M.sekaensis* sp. nov. **48** Nam Thaeng, Ubon Ratchathani Province, type locality of *M.ubonensis* sp. nov.

Unlike the northern region of Thailand, where diving beetle surveys have been carried out relatively many times (e.g., by Dr. William D. Shepard, Dr. Manfred A. Jäch, Dr. Herbert Zettel; pers. comm.), a large part of this country remained poorly explored, especially Isan (northeastern region) and the eastern regions. Our study detected three new *Microdytes* species from these unexplored areas. From the southern region no *Microdytes* species have been reported so far, although two species (*Microdyteselgae* Hendrich, Balke & Wewalka, 1995 and *M.pasiricus*) were recorded from Singapore, situated at the southern end of the Malay Peninsula (Wewalka 1997; Hendrich et al. 2004). Further new species can be expected from Thailand, particularly by collecting in unexplored provinces and repeated sampling at the microhabitat levels.

## Supplementary Material

XML Treatment for Microdytes eliasi

XML Treatment for Microdytes jeenthongi

XML Treatment for Microdytes maximiliani

XML Treatment for Microdytes sekaensis

XML Treatment for Microdytes ubonensis

XML Treatment for Microdytes akitai

XML Treatment for Microdytes balkei

XML Treatment for Microdytes dimorphus

XML Treatment for Microdytes franzi

XML Treatment for Microdytes gabrielae

XML Treatment for Microdytes heineri

XML Treatment for Microdytes maculatus

XML Treatment for Microdytes mariannae

XML Treatment for Microdytes menopausis

XML Treatment for Microdytes paoloi

XML Treatment for Microdytes pasiricus

XML Treatment for Microdytes pederzanii

XML Treatment for Microdytes rocchii

XML Treatment for Microdytes schoedli

XML Treatment for Microdytes schwendingeri

XML Treatment for Microdytes shepardi

XML Treatment for Microdytes shunichii

XML Treatment for Microdytes wewalkai

XML Treatment for Microdytes zetteli

XML Treatment for Microdytes schoenmanni
